# Traditional uses and emerging therapeutic potential of *Typha* angustifolia L.: insights into its phytochemistry, pharmacological activities and quality control

**DOI:** 10.3389/fphar.2025.1557136

**Published:** 2025-04-01

**Authors:** Junyu Liu, Ziyi Chen, Sian Tao, Xinyao Shu, Bingjie Shao, Xiaobo Zhang, Yang Yang, Xiaoli Wang

**Affiliations:** ^1^ College of Basic Medicine, Chengdu University of Traditional Chinese Medicine, Chengdu, Sichuan, China; ^2^ Institute of Traditional Chinese Medicine of Sichuan Academy of Chinese Medicine Sciences, Chengdu, Sichuan, China; ^3^ Department of Radiation Oncology, Yunnan Cancer Hospital, Kunming, Yunnan, China

**Keywords:** *Typha angustifolia* L., botany, traditional uses, phytochemistry, pharmacological activity, quality control

## Abstract

*Typha angustifolia* L. is a perennial marsh botanical drugs belonging to the genus *Typha* of the family Typhaceae, boasts a medicinal legacy spanning over 1900 years in China. Within traditional medicine, it is often used to treat a variety of bleeding disorders and gynecological diseases. *Typha angustifolia* contains various active components and metabolites including flavonoids, steroids, phenylpropanoids and organic acids. Over 94 compounds have been isolated and identified from *T. angustifolia*, demonstrating significant pharmacological activities such as anti-inflammatory, analgesic, anti-platelet aggregation, anti-atherosclerosis and anti-oxidation. In modern clinical practice, *T. angustifolia* is extensively utilized in treating dysmenorrhea, irregular menstruation, trauma bleeding, soft tissue contusion, hematochezia, hematuria and abnormal uterine bleeding. *Typha angustifolia* has a wide range of biological activities, making it a valuable resource for discovering potential drug candidates and developing new botanical supplements. This paper provides a comprehensive review of the research status of *T. angustifolia*, encompassing its botany, traditional uses, phytochemistry, pharmacological activity, and quality control, with the objective of enhancing our understanding of the application value and bioavailability of this traditional medicinal plant and offering a reference point for further research in this field.

## 1 Introduction

*Typha angustifolia* L., a renowned traditional herbal medicine, is derived from a perennial marsh plant belonging to the genus *Typha* of the family Typhaceae. This plant thrives in warm, humid swamps and shallow waters and is widely distributed in central and northeastern China, as well as in northern Asia, Europe, and North America (State Administration of TCM of the PR China, 1999). In ancient China, the yellow male inflorescences on the top of *T*. *angustifolia* were harvested in summer, dried, rolled, and sifted into powder for medicinal use, which has a history of more than 1900 years. Traditional ethnic medicine attributes hemostatic and diuretic properties to *T. angustifolia*, which finds widespread use in treating many diseases such as hematemesis, hemoptysis, traumatic bleeding, dysmenorrhea, thoracic and abdominal pain, swelling, urinary retention, hematochezia, and hematuria (Chinese pharmacopoeia committee, 2020; Nanjing University of TCM, 2006).

*Typha angustifolia* contains abundant bioactive metabolites, with 92 chemical metabolites currently isolated and identified, including flavonoids, steroids, alkaloids, organic acids, and volatile oils. Among them, flavonoids are considered the most representative active metabolites. Numerous *in vivo* and *in vitro* studies have demonstrated the wide range of biological activities exhibited by these compounds, including anti-inflammatory, analgesic, anti-platelet aggregation, anti-atherosclerosis, and anti-oxidation. In modern clinical practice, *T*. *angustifolia* is utilized in the treatment of benign prostatic hyperplasia, hyperlipidemia, and fundus vascular lesions (Li and Xu, 2018; Qin et al., 2017; Zhu, 2002). It is noteworthy that, as a marsh plant, *T*. *angustifolia* also plays a role in improving environmental water quality (Grebenshchykova et al., 2020; Nabuyanda et al., 2019; Reyes et al., 2013). It has a beneficial purification effect on heavy metal pollution and eutrophication and holds certain application value in urban sewage treatment (Zheng, 2018).

*Typha angustifolia* possesses high medicinal value and a broad spectrum of biological activities, presenting abundant resources for the discovery of promising drug candidates and the development of new plant supplements. However, current research on the active metabolites and mechanisms of *T*. *angustifolia* is insufficient, with a lack of mechanism-based pharmacological activity evaluation and *in vitro* and *in vivo* studies. Additionally, a systematic and effective quality control system has not been established, leading to the underutilization of its resources. Therefore, this paper reviews the research on *T*. *angustifolia* from various perspectives, including botany, traditional uses, phytochemistry, pharmacological activity, and quality control, by summarizing and discussing the research status, application prospects, and limitations, aims to provide theoretical support and guidance for the comprehensive development and utilization of this important medicinal plant resource.

## 2 Materials and methods

Following PRISMA guidelines for rigor and reproducibility, we conducted a systematic literature search with “*T. angustifolia*” as the central keyword, expanding it iteratively to include synonymous terms, related pharmacological applications, and therapeutic outcomes. Boolean operators were used to combine search strings focusing on both plant identity and research scope. We examined a wide range of academic sources from international databases such as PubMed, ScienceDirect, Web of Science, CNKI and Baidu Scholar, and supplemented these with manual searches of the Chinese Pharmacopoeia and historical texts. All publications in peer reviewed journals until December 2024 were considered.

## 3 Botany

**FIGURE 1:**
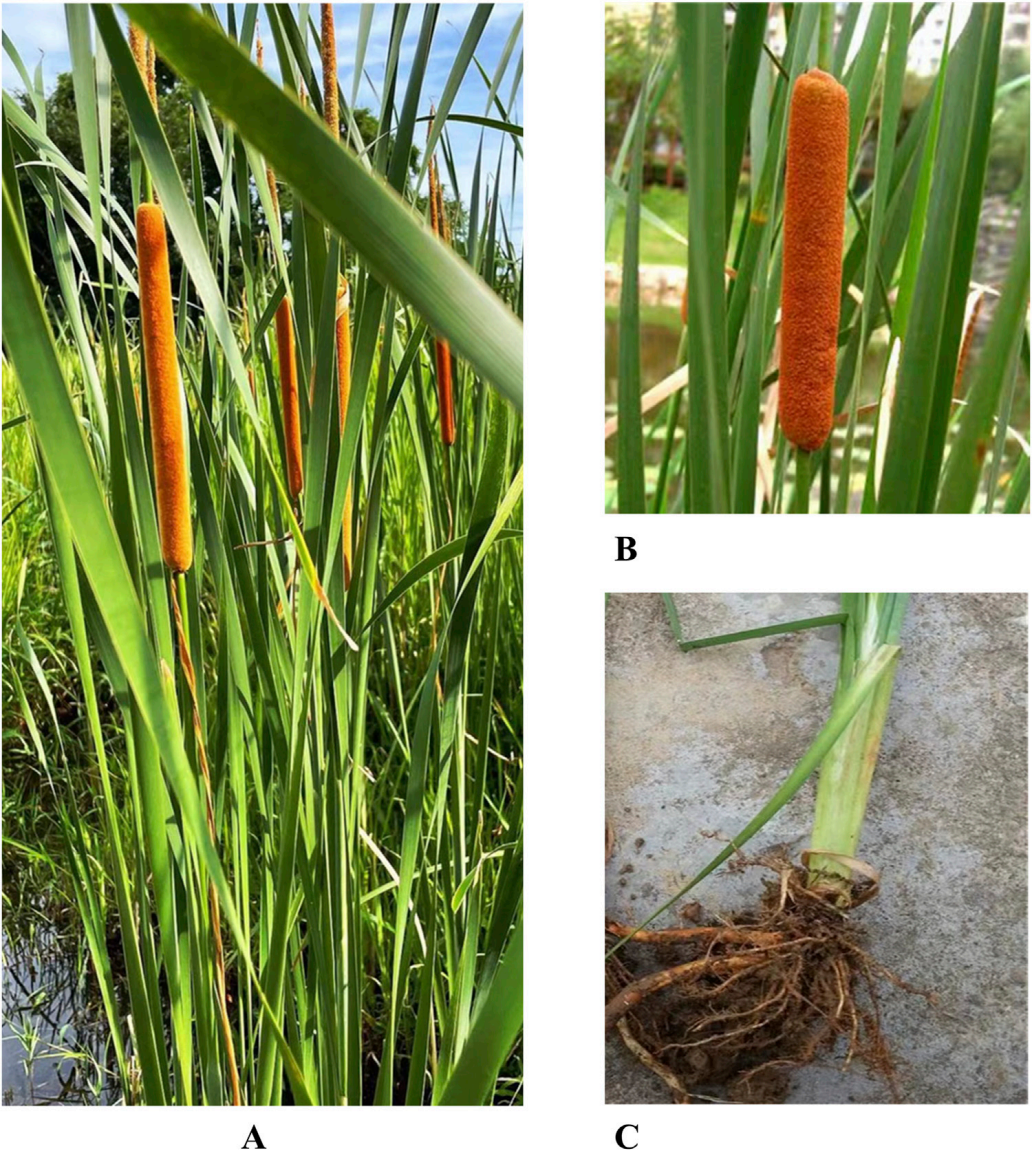
*Typha angustifolia*: **(A)** whole plant **(B)** inflorescences **(C)** stem and rhizome.

*Typha angustifolia* is a perennial botanical drugs, typically reaching a height of 1.5–3 m. The rhizomes of the plant are milky yellow in color. The stem is erect and stout in the aboveground portion, gradually tapering as it ascends. The leaves are strap-shaped, measuring 40–70 cm in length and 0.4–0.9 cm in width. They possess a smooth and hairless surface, and the upper part of the leaves is flat, while the lower part’s abdomen is slightly concave. The back of the leaves gradually rises and protrudes outward, resulting in a semicircular transverse section, the base of the sheath expands and grows around the part of the stem. The inflorescences are typically spaced around 2.5–6.9 cm apart, with the male inflorescences measuring approximately 2.7–9.2 cm in length. The central axis of the inflorescences is white, curved, and covered with soft hairs. From the base of the inflorescences, there are 1-3 bracteoles slightly wider than the leaves. The lower part consists of the female flowers, measuring about 15–30 cm in length, with a bract also growing slightly wider than the leaf. The male flower is usually composed of three stamens, with anthers about 2 mm long and containing two chambers, and the overall shape is striped, with distinct nearly spherical pollen grains. The filaments are short, and the flower is united at the base into a short stalk, measuring about 2–3 mm in length. The stigma is spoon-shaped, curved outward, about 1.3–1.8 mm in length. The ovary is fusiform, exhibiting brown spots on the surface, and approximately 1 mm in length. The stalk at the root of the ovary is thinner, about 5 mm in length. The sterile female ovary is 1–1.2 mm long, conical in shape, with the uppermost portion yellowish brown and short-pointed, displaying undeveloped stigmas; the base of the ovary is frequently white, adorned with filamentous hairs that extend upward, slightly shorter than the stigma. The fruit type is a nut, the shape is oval, measuring approximately 1.5 mm in length. The pericarp has long brown spots, and the seeds are dark brown, about 1–1.2 mm in length. The florescence of *T. angustifolia* is from June to August, and the fruiting period is from August to September (Zhang, 1998; State Administration of TCM of the PR China, 1999; Nanjing University of TCM, 2006). The whole plant and inflorescence are shown in Figure 1.

## 4 Traditional uses

*Typha angustifolia*, known as “Pu Huang” in Chinese, refers to an botanical drug renowned for its styptic and blood-stasis properties. Traditional Chinese Medicine extensively utilizes *T*. *angustifolia* in the treatment of dysmenorrhea, irregular menstruation, abdominal pain, trauma bleeding, soft tissue contusion, hematochezia, hematuria, and functional uterine bleeding (Nanjing University of TCM, 2006). Beyond its clinical applications, *T*. *angustifolia* also serves as a vital aquatic economic plant. Its leaf bases and rhizome apices are edible vegetables, while its stems and leaves are utilized for weaving mats and other crafts, as well as in paper manufacturing, the female flower can be employed as filling for pillow cores and cushions (Zhao and Xu, 2007).

The first documented of *T*. *angustifolia* was in the *Shen Nong Ben Cao Jing*, the earliest Chinese treatise on materia medica which over 1,900 years ago (Sun, 1963). It describes *T*. *angustifolia* as a Chinese herbal medicine known for its diuretic, analgesic, and hemostatic effects. In the Tang Dynasty, the herbal book *Yao Xing Lun* records that *T*. *angustifolia* had a prominent hemostatic effect and could be used to treat a variety of bleeding diseases, including abnormal uterine bleeding, nasal mucosal bleeding and hematuria (Zhen, 2006). In the Classic Materia Medica, *Ri Hua Zi Ben Cao* in the Song Dynasty, records that *T*. *angustifolia* can be used to treat epigastric pain, dysmenorrhea, irregular menstruation, uroschesisn and hematochezia (Ri, 2005). *Ben Cao Gang Mu*, a botanical monograph of the Ming Dynasty, records that *T*. *angustifolia* had a good analgesic effect, which could be used to relieve various types of pathologic pain, such as chest pain, heartache, stomachache and bellyache (Li, 1957). The same period’s Materia Medica *Ben Cao Jing Shu* also had a similar record, and it was believed that this analgesic effect of *T*. *angustifolia* might be related to its influence on the blood circulation system (Miao, 2011). The traditional uses of *T*. *angustifolia* is more detailed in the Materia Medica classics of the Qing Dynasty. It is recorded in *Ben Cao Chong Yuan* that *T*. *angustifolia* can be used to treat a variety of hemorrhagic diseases such as hematuria, traumatic bleeding and digestive tract bleeding (Zhang, 1983). Notably, *T*. *angustifolia* is effective in alleviating pain symptoms commonly associated with bleeding. *Ben Cao Bei Yao* was the first to suggest that the therapeutic effect of *T*. *angustifolia* could be enhanced through high-temperature roasting, and it also reported that *T*. *angustifolia* can be used to treat soft tissue contusion, menorrhagia, spermatogenesis and urinary retention (Wang, 2005). In addition, *T*. *angustifolia* also plays an important role in the treatment of some digestive diseases, such as flatulence, digestive dysfunction and enteritis. This is confirmed by the relevant records in *Ben Cao Cong Xin* and *Ben Cao Bian Du* (Wu, 2013; Zhang, 1936). The ancient Classic Medica books on *T*. *angustifolia* and their traditional uses are shown in Table 1.

**TABLE 1:** Traditional uses of *Typha angustifolia*.

Dynasty of ancient China	Classic medica books	Traditional use	References
The Eastern Han Dynasty (A. D. 184)	*Shen Nong Ben Cao Jing*	Used in the treatment of hypogastralgia, cystodynia, dysfunctional urination and blood stasis	Sun (1963)
The Tang Dynasty (A. D. 653)	*Yao Xing Lun*	Used as a hemostatic to treat abnormal uterine bleeding, nasal mucosal bleeding, hematochezia and hematuria	Zhen (2006)
The Song Dynasty (A. D. 908)	*Ri Hua Zi Ben Cao*	Treat epigastric pain, dysmenorrhea, irregular menstruation, uroschesisn and hematochezia	Ri (2005)
The Ming Dynasty (A. D. 1,552)	*Ben Cao Gang Mu*	Relieve heartache, epigastric pain, stomachache and bellyache	Li (1957)
The Ming Dynasty (A. D. 1,625)	*Ben Cao Jing Shu*	For the treatment of blood stasis, stuffiness and palpitation	Miao (2011)
The Qing Dynasty (A. D. 1,674)	*Ben Cao Chong Yuan*	Used to treat bellyache, precordialgia, hematuria, traumatic bleeding, dysfunctional urination and blood stasis	Zhang (1983)
The Qing Dynasty (A. D. 1,694)	*Ben Cao Bei Yao*	Used in the treatment of menorrhagia, spermatorrhea, uroschesisn and gastrointestinal bleeding	Wang (2005)
The Qing Dynasty (A. D. 1757)	*Ben Cao Cong Xin*	For the treatment of epigastric pain, digestive dysfunction and bleeding and swelling caused by trauma	Wu (2013)
The Qing Dynasty (A. D. 1887)	*Ben Cao Bian Du*	Used to treat blood stasis, dyspeptic abdominalgia and heartache	Zhang (1936)

Moreover, *T*. *angustifolia* is also a crucial component in over 10 listed traditional Chinese medicine prescription preparations, including Jinggu Dieda pills, Fuke Zhixue Ling, Mo Luo dan and Hexue Mingmu Pian. These formulations are utilized in treating conditions such as soft tissue contusion, functional uterine bleeding, chronic atrophic gastritis, and fundus vascular diseases (Li and Xu, 2018; Qi et al., 2022; Xue et al., 2023). All the formulated preparation of Traditional Chinese Medicine containing *T*. *angustifolia* and their clinical applications are shown in Table 2.

**TABLE 2:** Clinical application of formulated preparation of Traditional Chinese Medicine of *Typha angustifolia*.

Name	Formulation	National drug approval number	Clinical application	Ref.
Jingu Dieda Wan	Pill	Z20027151	Traumatic bruise, fracture and tendon injury, soft tissue injury	National Medical Products Administration (2002)
Zhentong Huoluo Ding	Tincture	Z20184036	Chronic soft tissue injury, arthritis, scapulohumeral periarthritis, cervical spondylosis, hyperosteogeny, sciatica	National Medical Products Administration (2002)
Suantong Penwu	Spray	Z20026569	Sprains, fatigue injuries, musculoskeletal soreness	National Medical Products Administration (2002)
Hexue Mingmu Pian	Tablet	Z20073062	Fundus vascular disease, retinal hemorrhage, blurred vision	Chinese pharmacopoeia committee, 1998
Fuke Huisheng Wan	Pill	Z12020076	Irregular menstruation, dysmenorrhea, subinvolution of uterus	Medical department pharmacopoeia committee, 1998
Fuke Zhixueling	Capsule	Z22023205	Dysfunctional uterine bleeding, menorrhagia	Medical department pharmacopoeia committee, 1998
Puhuang Pian	Tablet	Z53021387	Hyperlipidemia, hypercholesterolemia	Medical department pharmacopoeia committee, 1998
Huisheng Koufu Ye	Syrup	Z20025042	Primary liver cancer, lung cancer	National Medical Products Administration (2002)
Gongxue Ting Keli	Granule	Z23022074	Menorrhagia, abnormal uterine bleeding	Medical department pharmacopoeia committee, 1998
Shixiang Zhitong Wan	Pill	Z45020153	Epigastric pain, stomachache, bellyache, hypochondriac pain	Chinese pharmacopoeia committee, 2020
Mo Luo Dan	Pill	Z13021324	Chronic atrophic gastritis, stomachache, gastroesophageal reflux, gastric dysmotility	Medical department pharmacopoeia committee, 1998
Jiechang Ning	Suppository	Z10890022	Chronic bacillary dysentery, chronic colitis, ulcerative colitis	Medical department pharmacopoeia committee, 1998
Naoshuan Tong Jiaonang	Capsule	Z20040093	Acute ischemic stroke, the rehabilitation stage of stroke	Chinese pharmacopoeia committee, 2020

## 5 Phytochemistry

**FIGURE 2:**
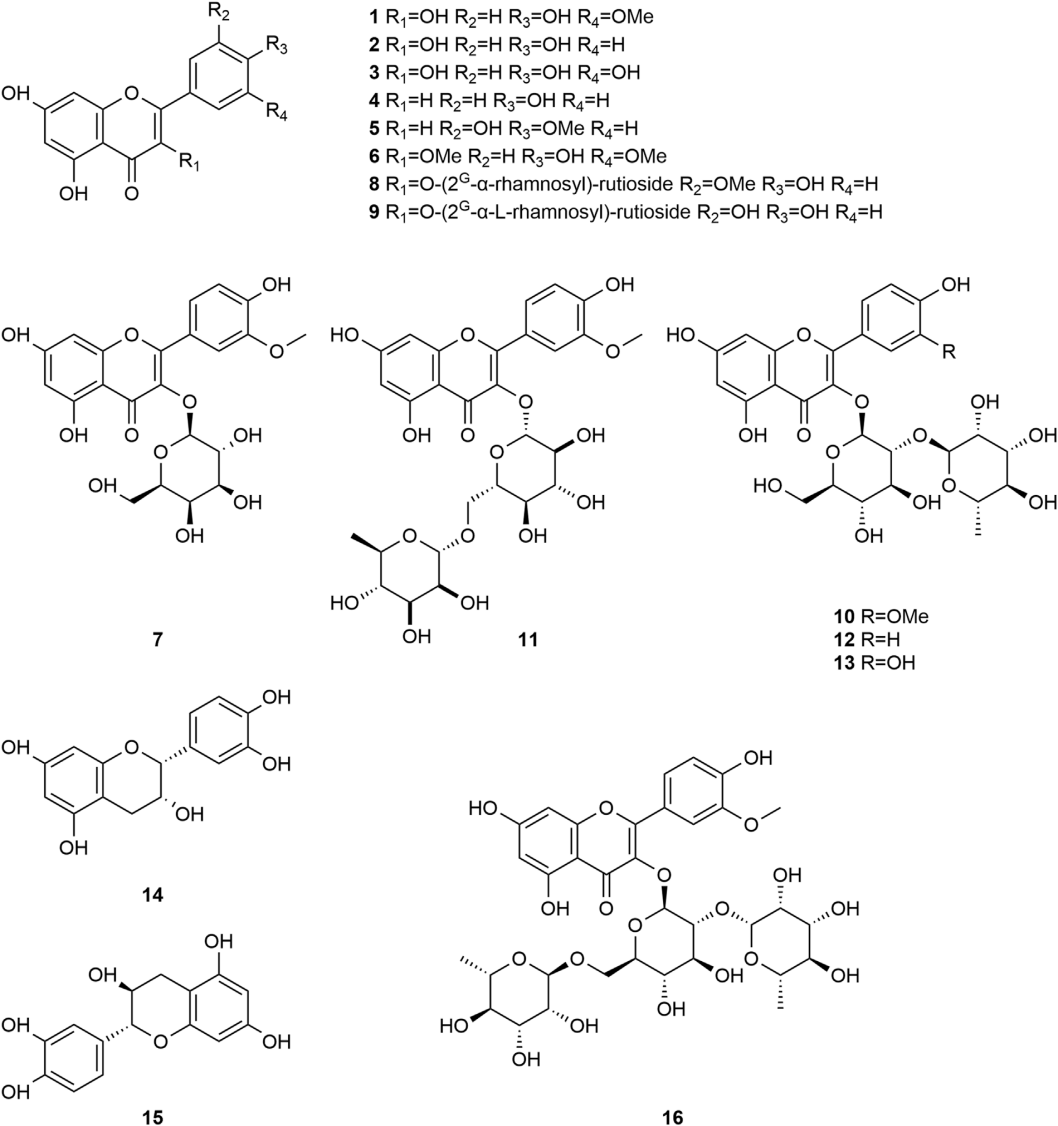
Structure of flavonoids from *Typha angustifolia* L.

**FIGURE 3:**
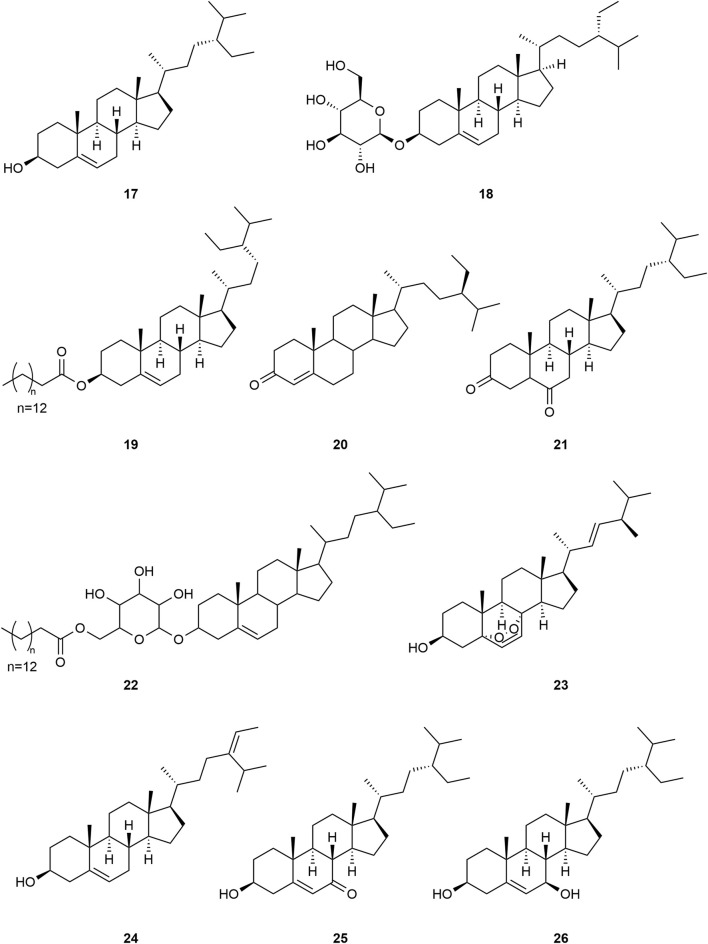
Structure of steroids from *Typha angustifolia* L.

**FIGURE 4:**
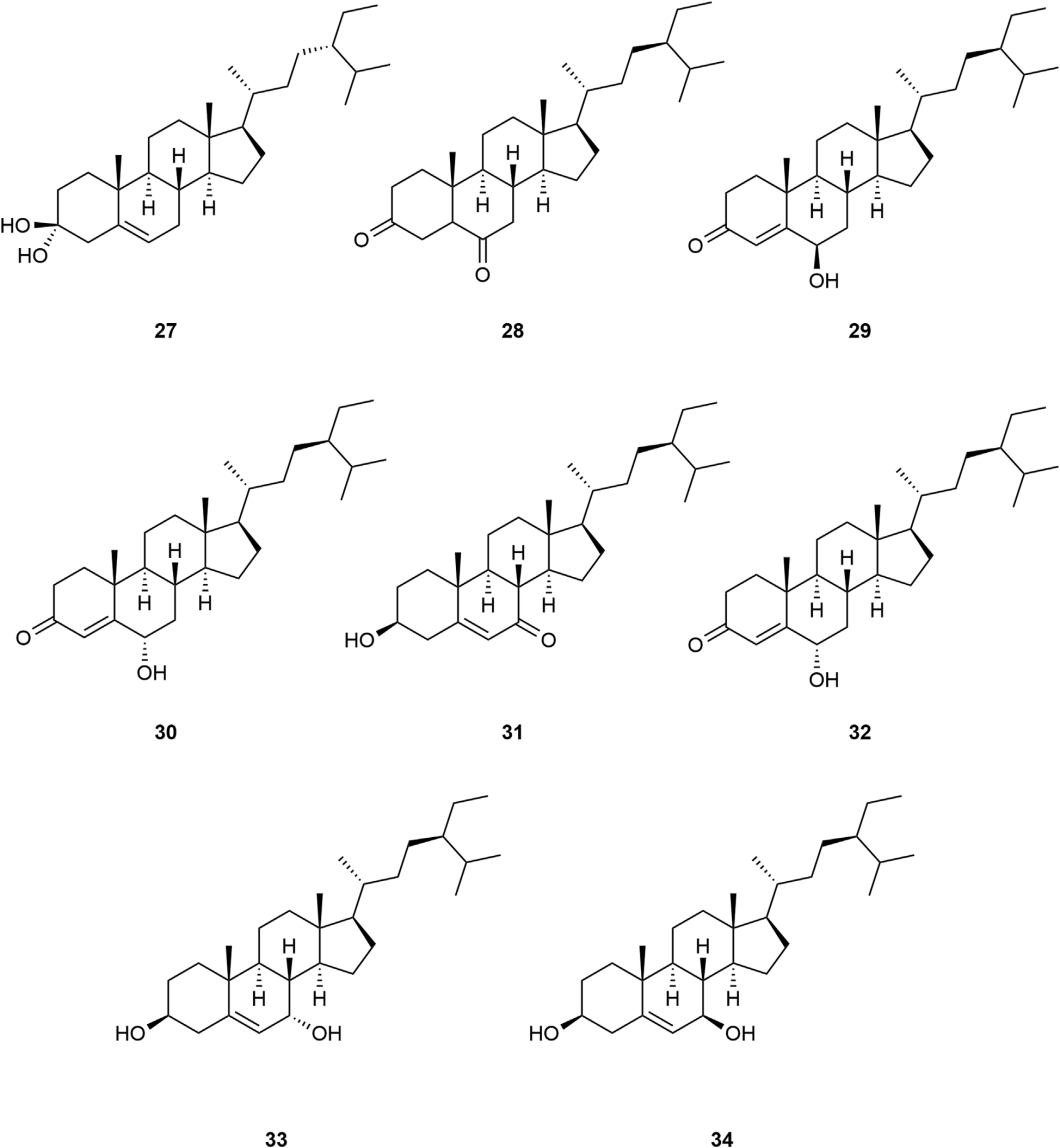
Structure of Steroids from *Typha angustifolia* L.

**FIGURE 5:**
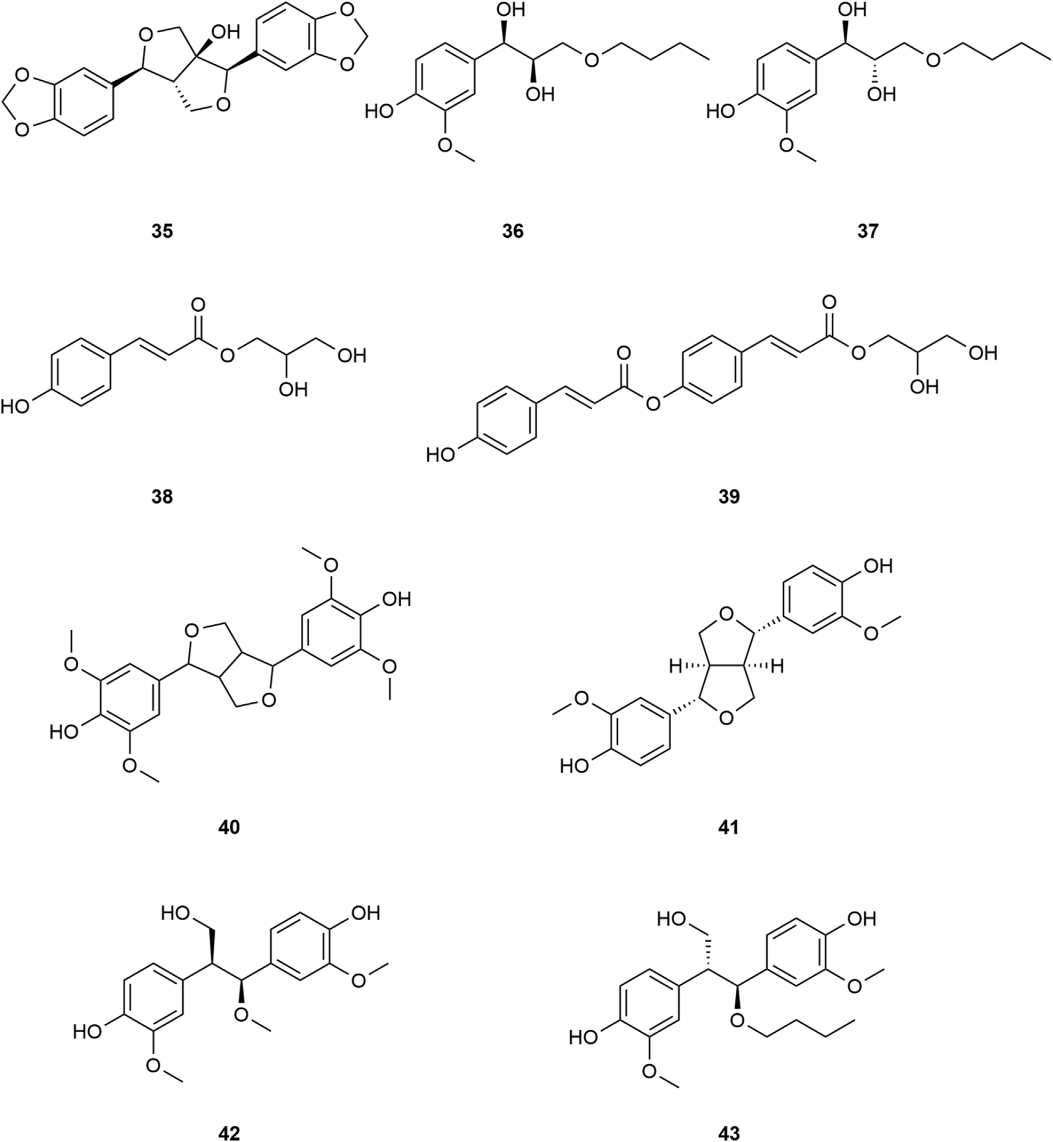
Structure of phenylpropanoids from *Typha angustifolia* L.

**FIGURE 6:**
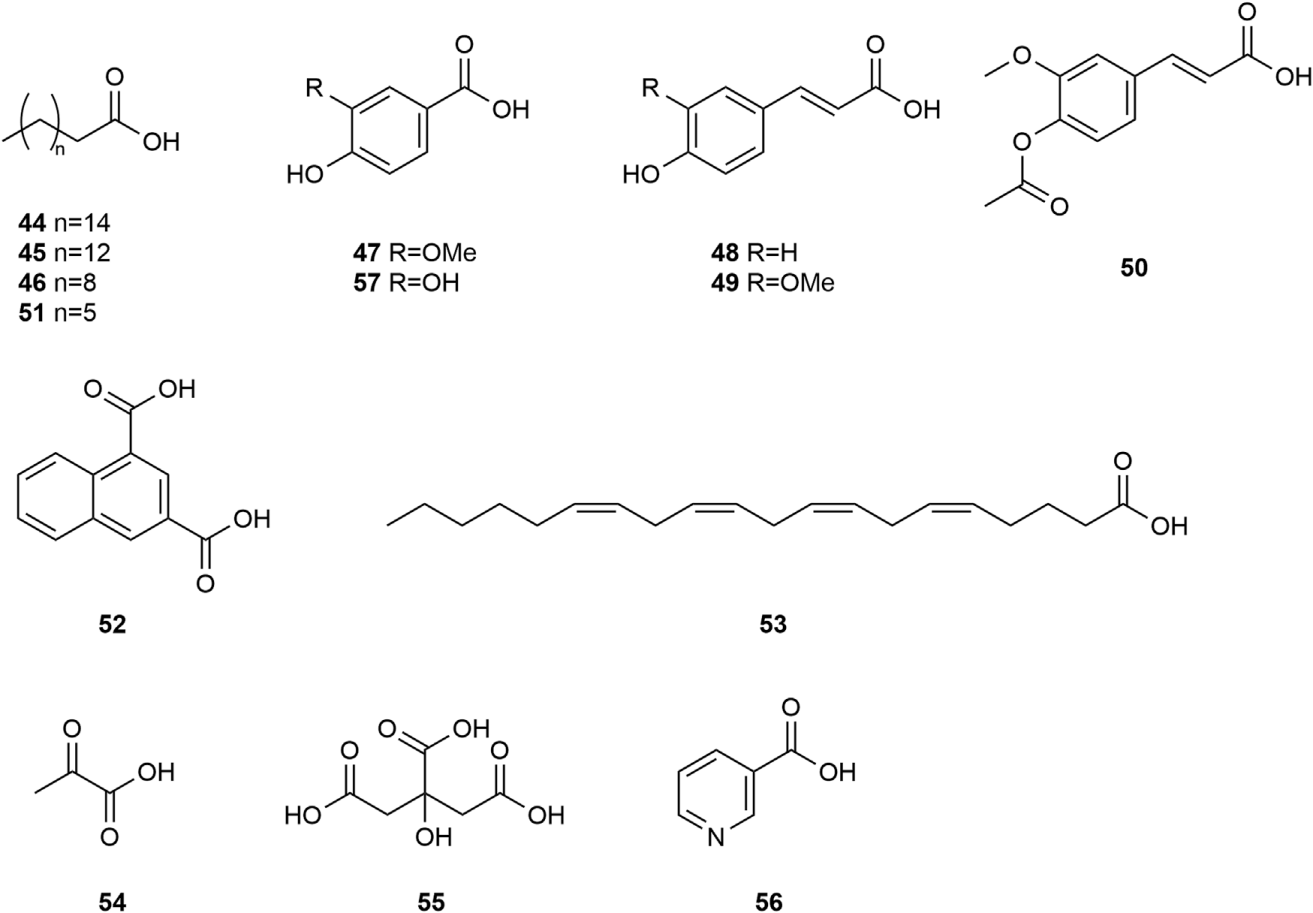
Structure of Organic acids from *Typha angustifolia* L.

**FIGURE 7:**
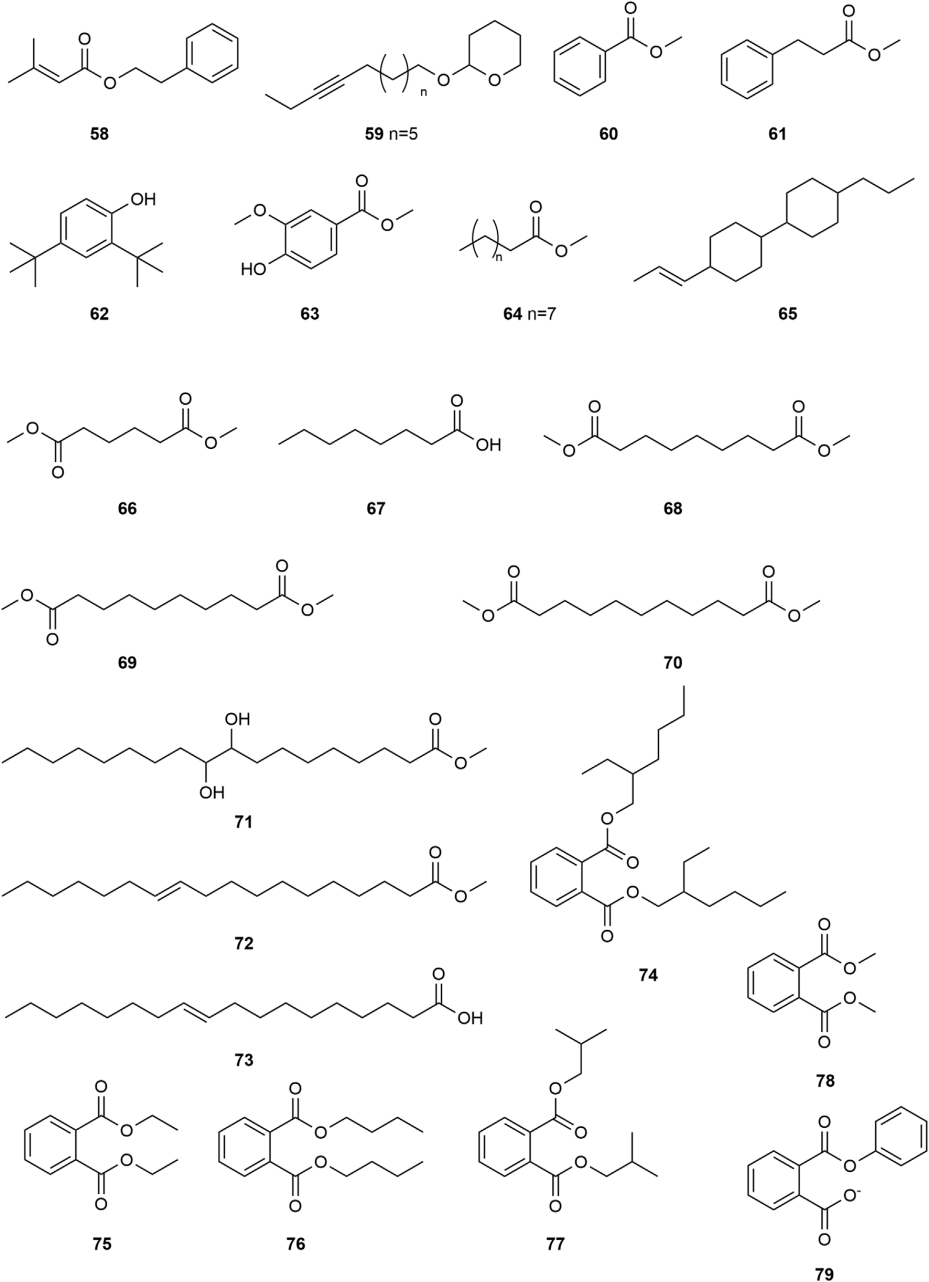
Structure of Volatile oil from *Typha angustifolia* L.

**FIGURE 8:**
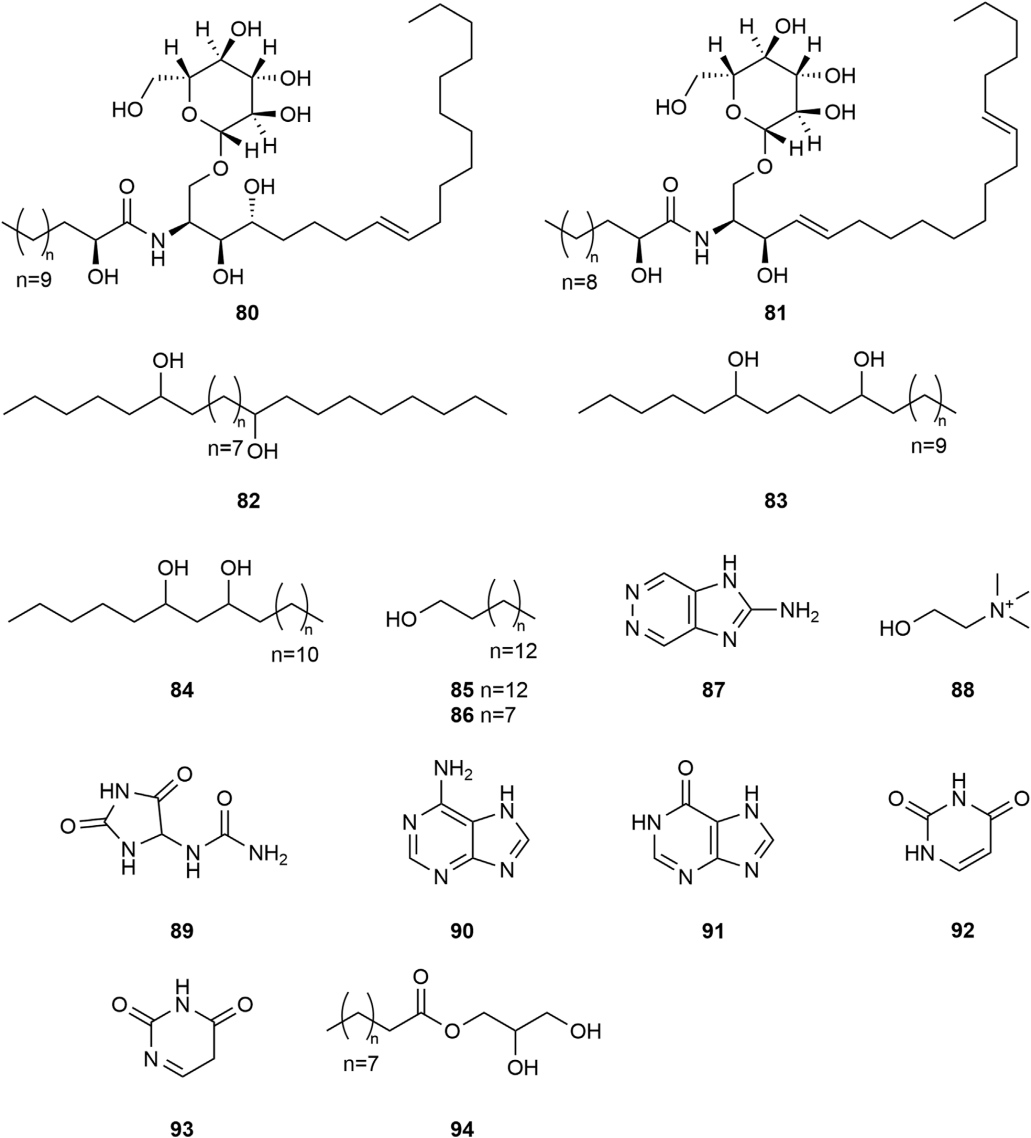
Structure of other compounds from *Typha angustifolia* L.

**FIGURE 9:**
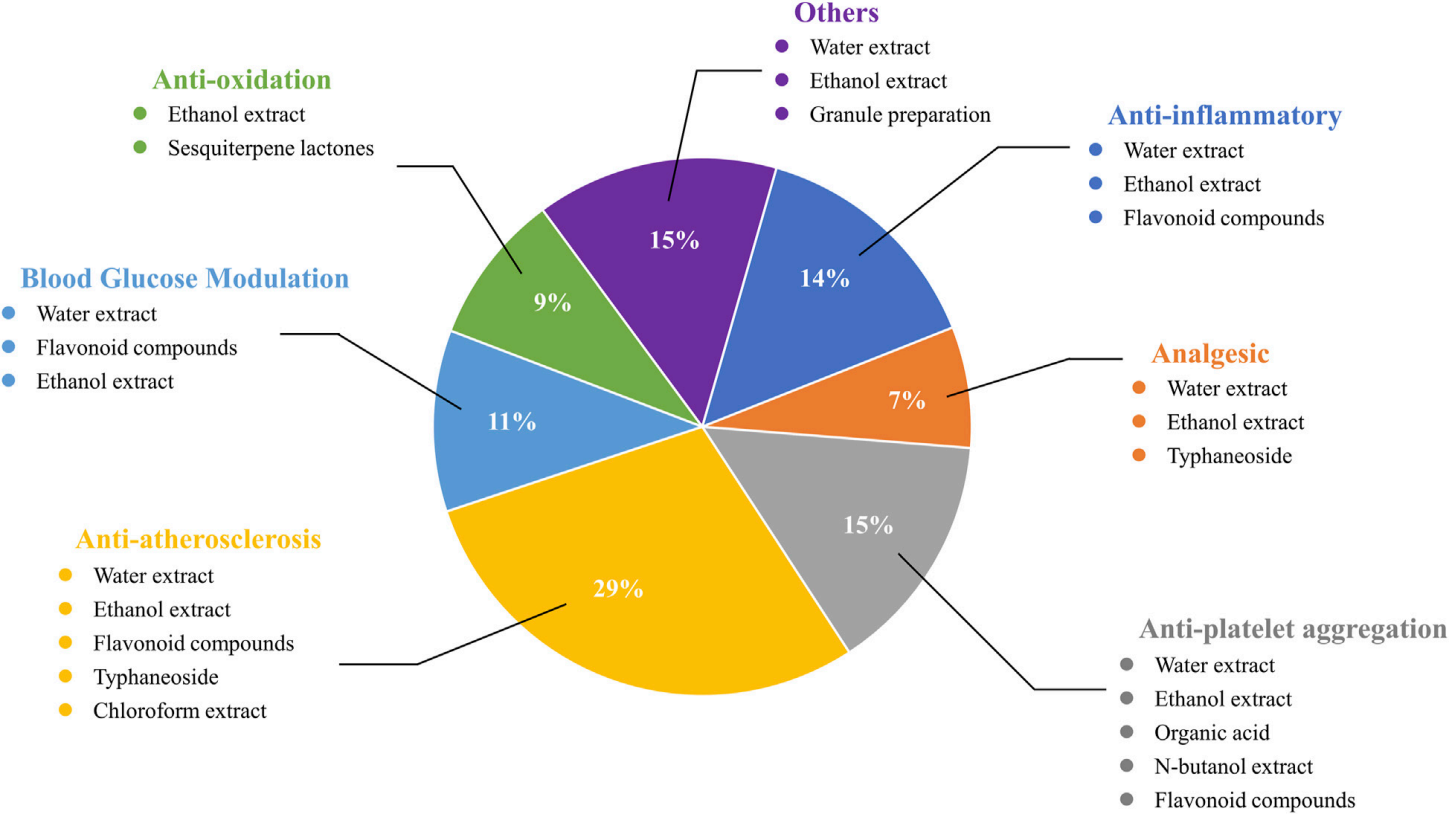
Biological activity of *Typha angustifolia* L.

*Typha angustifolia* contains a rich array of chemical metabolites, with 94 compounds having been isolated and identified thus far. These compounds include flavonoids (**1–16**), steroids (**17–34**), phenylpropanoids (**35–43**), Organic acid (**44–57**), volatile oil (**58–79**) and other compounds (**80–94**). Phytochemical studies indicate that the most active metabolites are found in the pollen of *T. angustifolia*, with flavonoids being the predominant constituent. The names, categories and corresponding structures of these compounds are summarized in Table 3 and Figures 2–9.

**TABLE 3:** Chemical metabolites from *Typha angustifolia*.

Classification	No.	Name	Ref
Flavonoid	1	Isorhamnetin	Gao and Tian (2001)
	2	Kaempferol	Gao and Tian (2001)
	3	Quercetin	Gao and Tian (2001)
	4	Naringenin	Chen et al. (2008)
	5	Quercetin-3-O-rutinose	Liao et al. (1990)
	6	3.3′-Dimethylquercetin	Liao et al. (1990)
	7	Cacticin	Liao et al. (1990)
	8	Isorhamnetin-3-O-(2^G^-α-rhamnosyl)-rutioside	Chen et al. (2019)
	9	Quercetin-3-O-(2^G^-α-L-rhamnosyl)-rutioside	Chen et al. (2019)
	10	Isorhamnetin-3-O-neohesperidin	Zeng et al. (2018)
	11	Narcissin	Wang et al. (1998)
	12	Kaempferol-3-O-neohesperidoside	Wang et al. (1998)
	13	Quercetin-3-O-neohesperidoside	Wang et al. (1998)
	14	Epicatechin	Liu et al. (1998)
	15	Catechin	Liu et al. (1998)
	16	Typhaneoside	Liu et al. (1998)
Steroid	17	β-Sitosterol	Chen (1990)
	18	Daucosterol	Chen (1990)
	19	Sitosteryl palmitate	Chen (1990)
	20	β-sitostenone	Kong et al. (2011)
	21	Stigmastane-3,6-dione	Kong et al. (2011)
	22	Daucosterol 6′-O-palmitate	Chen et al. (2015)
	23	Peroxyergosterol	Chen et al. (2019)
	24	Isofucosterol	Takatsuto (1997)
	25	7-oxositosterol	Monaco et al. (1990)
	26	7β-hydroxysitosterol	Monaco et al. (1990)
	27	7α-hydroxysitosterol	Monaco et al. (1990)
	28	Stigmastan-3-6-dione	Monaco et al. (1990)
	29	Stigmast-4-en-6β-ol-3-one	Monaco et al. (1990)
	30	Stigmast-4-en-6α-ol-3-one	Monaco et al. (1990)
	31	3β-hydroxystigmast-5-en-7-one	Monaco et al. (1990)
	32	6α-hydroxystigmast-4-en-3-one	Monaco et al. (1990)
	33	Stigmast-5-ene-3β,7α-diol	Monaco et al. (1990)
	34	Stigmast-5- ene-3β,7β-diol	Monaco et al. (1990)
Phenylpropanoid	35	Paulownin	Guo et al. (2019)
	36	9-n-butyl-guaiacylglycerol	Guo et al. (2019)
	37	9-n-butyl-isoguaiacylglycerol	Guo et al. (2019)
	38	1-O-p-coumaroylglycerol	Guo et al. (2019)
	39	1-O-di-p-coumaroylglycerol	Guo et al. (2019)
	40	Syringaresinol	Guo et al. (2019)
	41	Pinoresinol	Guo et al. (2019)
	42	Threo-2,3-bis-(4-hydroxy-3-methoxyphenyl)-3-methoxypropanol	Guo et al. (2019)
	43	Erythro-2,3-bis(4-hydroxy-3-methoxyphenyl)-3-butoxypro pan-1-ol	Guo et al. (2019)
Organic acid	44	Palmitic Acid	Chen., 1990
	45	Cerotinic acid	Chen., 1990
	46	Octadecanoic acid	Chen et al. (2008)
	47	Vanillic acid	Mo et al. (2009)
	48	4-Hydroxycinnamic acid	Feng et al. (2012b)
	49	Ferulic acid	Feng et al. (2012b)
	50	4-Acetoxy-3-methoxycinnamic acid	Feng et al. (2012b)
	51	Lauric acid	Chen et al. (2015)
	52	Typhic acid	Xu et al. (1987)
	53	Arachidonic acid	Xu et al. (1987)
	54	Pyruvic Acid	Zhang et al. (2003)
	55	Citric Acid	Zhang et al. (2003)
	56	Nicotinic acid	Feng et al. (2014a)
	57	Protocatechuic acid	Feng et al. (2014a)
Volatile oil	58	Phenethyl senecioate	Chen et al. (2015)
	59	Tetrahydro-2-(12-pentadecynyloxy)-2H-pyran	Chen et al. (2015)
	60	Methyl benzoate	Chen et al. (2015)
	61	Methyl hydrocinnamate	Chen et al. (2015)
	62	2, 4-di-tert-butylphenol	Chen et al. (2015)
	63	Methyl vanillate	Chen et al. (2015)
	64	Methyl hexadecanoate	Chen et al. (2015)
	65	1.1′-bicyclohexy	Chen et al. (2019)
	66	Dimethyl adipate	Chen et al. (2019)
	67	Octanoic acid	Chen et al. (2019)
	68	Dimethyl azelate	Chen et al. (2019)
	69	Dimethyl sebacate	Chen et al. (2019)
	70	Dimethyl undecanedioate	Chen et al. (2019)
	71	Methyl 9,10-dihydroxyoctadecanoate	Chen et al. (2019)
	72	Methyl vaccenate	Chen et al. (2019)
	73	10-Octadecenoic acid	Chen et al. (2019)
	74	bis (2-ethylhexyl) phthalate	Chen et al. (2019)
	75	Diethyl phthalate	Chen et al. (2019)
	76	Dibutyl phthalate	Chen et al. (2019)
	77	Diisobutyl phthalate	Chen et al. (2019)
	78	Dimethyl phthalate	Chen et al. (2019)
	79	Phenyl phthalate	Chen et al. (2019)
Others	80	1-O-(β-D-glucopyranosyloxy)-(2S,3S,4R,8Z)-2-[(2′R)-2′-ydroxytricosanoylamino]-8-nonadecene-3,4-diol	Tao et al. (2010)
	81	1-O-(β-D-glucopyranosyloxy)-(2S,3R,4E,8Z)-2-[(2′R)-2′-hydroxynonadecanoylamino]-4.13 -nonadecene-3-diol	Tao et al. (2010)
	82	6,21-Nonacosanediol	Chen et al. (2019)
	83	6,10-Nonacosanediol	Chen et al. (2019)
	84	6,8-Nonacosanediol	Chen et al. (2019)
	85	1-Hexacosanol	Chen et al. (2019)
	86	Hexadecanol	Chen et al. (2019)
	87	Zarzissine	Feng et al. (2014a)
	88	Choline	Feng et al. (2014a)
	89	Allantoin	Chen et al. (2015)
	90	Adenine	Chen et al. (2015)
	91	Hypoxanthine	Chen et al. (2015)
	92	Uracil	Chen et al. (2015)
	93	2.4(3H,5H)-Pyrimidinedione	Feng et al. (2014)
	94	2.3- Dihydroxypropyl hexadecanoate	Chen et al. (2015)

### 5.1 Flavonoids

Flavonoid compounds are abundant in *T*. *angustifolia*, and are considered as the basis of many pharmacological activities such as anti-inflammation, analgesia and anti-hyperlipidemic. At present, a total of 16 flavonoids (**1–16**) have been isolated and identified from *T*. *angustifolia*, including Isorhamnetin **(1)**, Kaempferol **(2)**, Quercetin **(3)**, Naringenin **(4)**, Quercetin-3-O-rutinose **(5)**, 3.3′-Dimethylquercetin **(6)**, Cacticin **(7)**, Isorhamnetin-3-O-(2G-α-rhamnosyl)-rutioside **(8)**, Quercetin-3-O-(2G-α-L-rhamnosyl)-rutioside **(9)**, Isorhamnetin-3-O-neohesperidin **(10)**, Narcissin **(11)**, Kaempferol-3-O-neohesperidoside **(12)**, Quercetin-3-O-neohesperidoside **(13)**, Picatechin **(14)**, Catechin **(15)** and Typhaneoside **(16).** Moreover, the compounds sorhamnetin-3-O-neohesperidin **(10)** and Typhaneoside **(16)** have also been reported to have significant antioxidant activities and promote the proliferation of human umbilical vein endothelial cells *in vitro* (Chen et al., 2017). These compounds and their corresponding structures are shown in Table 3 and Figure 2.

### 5.2 Steroids

A total of 18 steroid compounds (**17–34**) were isolated from *T*. *angustifolia*, most of which were cyclopentane-polyhydrophenanthrene as the structural framework. Monaco et al. (1990) isolated several novel sterols with ergostane skeleton from *T*. *angustifolia*, and the compounds 7β-hydroxysitosterol **(26)** and 7α-hydroxysitosterol **(27)** were isolated from this plant for the first time. These compounds are β-Sitosterol **(17)**, Daucosterol **(18)**, Sitosteryl palmitate **(19)**, β-sitostenone **(20)**, Stigmastane-3,6-dione **(21)**, Daucosterol 6′-O-palmitate **(22)**, Peroxyergosterol **(23)**, Isofucosterol **(24)**, 7-oxositosterol **(25)**, 7β-hydroxysitosterol **(26)**, 7α-hydroxysitosterol **(27)**, Stigmastan-3-6-dione **(28)**, Stigmast-4-en-6β-ol-3-one **(29)**, Stigmast-4-en-6α-ol-3-one **(30)**, 3β-hydroxystigmast-5-en-7-one **(31)**, 6α-hydroxystigmast-4-en-3-one **(32)**, Stigmast-5-ene-3β,7α-diol **(33)** and Stigmast-5- ene-3β,7β-diol **(34)**. These compounds and their corresponding structures are shown in Table 3 and Figures 3, 4.

### 5.3 Phenylpropanoids

The phenylpropanoids in *T*. *angustifolia* are mainly divided into coumarins and lignin, which exhibit distinct structural characteristics. Coumarins are characterized by a benzene ring fused to an alpha-pyrone ring, while lignin compounds typically consist of phenylpropane units linked by various types of bonds, forming a complex polymer structure. At present, nine compounds (**35–43**) have been isolated, which are respectively aulownin **(35)**, 9-n-butyl-guaiacylglycerol **(36)**, 9-n-butyl-isoguaiacylglycerol **(37)**, 1-O-p-coumaroylglycerol **(38)**, 1-O-di-p-coumaroylglycerol **(39)**, Syringaresinol **(40)**, Pinoresinol **(41)**, Threo-2,3-bis-(4-hydroxy-3-methoxyphenyl)-3-methoxypropanol **(42)** and Erythro-2,3-bis(4-hydroxy-3-methoxyphenyl)-3-butoxypro pan-1-ol **(43)**. These compounds and their corresponding structures are shown in Table 3 and Figure 5.

### 5.4 Organic acids

At present, fourteen organic acid compounds (**44–57**) were isolated from *T*. *angustifolia*, predominantly consisting of unsaturated organic acids and long-chain fatty acids. These compounds are Palmitic Acid **(44)**, Cerotinic acid **(45)**, Octadecanoic acid **(46)**, Vanillic acid **(47)**, 4-Hydroxycinnamic acid **(48)**, Ferulic acid **(49)**, 4-Acetoxy-3-methoxycinnamic acid **(50)**, Lauric acid **(51)**, Typhic acid **(52)**, Arachidonic acid **(53)**, Pyruvic Acid **(54)**, Citric Acid **(55)**, Nicotinic acid **(56)** and Protocatechuic acid **(57)**. These compounds, such as Ferulic acid **(49)** and Citric acid **(55)**, have shown promising anti-oxidant and anti-inflammatory properties in recent studies, and also contribute to the diverse chemical composition of *T. angustifolia*, offering potential pharmaceutical applications in various health conditions (Liu et al., 2022; Wu et al., 2019). These compounds and their corresponding structures are shown in Table 3 and Figure 6.

### 5.5 Volatile oil

The volatile oil content in *T. angustifolia* is relatively abundant. Currently, 23 compounds were isolated and identified from *T*. *angustifolia*, including Phenethyl senecioate **(58)**, Tetrahydro-2-(12-pentadecynyloxy)-2H-pyran **(59)**, Methyl benzoate **(60)**, Methyl hydrocinnamate **(61)**, 2, 4-di-tert-butylphenol **(62)**, Methyl vanillate **(63)**, Methyl hexadecanoate **(64)**, 1,1′-bicyclohexy **(65)**, Dimethyl adipate **(66)**, Octanoic acid **(67)**, Dimethyl azelate **(68)**, Dimethyl sebacate **(69)**, Dimethyl undecanedioate **(70)**, Methyl 9,10-dihydroxyoctadecanoate **(71)**, Methyl vaccinate **(72)**, 10-Octadecenoic acid **(73)**, bis(2-ethylhexyl) phthalate **(74)**, Diethyl phthalate **(75)**, Dibutyl phthalate **(76)**, Diisobutyl phthalate **(77)**, Dimethyl phthalate **(78)** and Phenyl phthalate **(79)**. The volatile oil metabolite is crucial for evaluating *T*. *angustifolia* quality. Biological activity studies indicate that many of the aforementioned metabolites exhibit strong biological activity. For example, Dimethyl phthalate **(78)** is commonly used in mosquito repellent production due to its exceptional adhesion and water resistance (Li et al., 2006). These compounds and their corresponding structures are shown in Table 3 and Figure 7.

### 5.6 Others

Tao et al. (2010) first reported two new cerebrosides in *T*. *angustifolia*, 1-O-(β-D-glucopyranosyloxy)-(2S,3S,4R, 8Z)-2-[(2′R)-2′-ydroxytricosanoylamino]-8-nonadecene-3,4-diol **(80)** and 1-O-(β-D-glucopyranosyloxy)-(2S,3R,4E, 8Z)-2-[(2′R)-2′-hydroxynonadecanoylamino]-4.13 -nonadecene-3-diol **(81)**, and found that they could inhibited vascular smooth muscle cell proliferation in a dose-dependent manner. Additionally, *T*. *angustifolia* also contains some aliphatic hydrocarbons and alkaloids, such as 6,21-Nonacosanediol **(82)**, 6,10-Nonacosanediol **(83)**, 6,8-Nonacosanediol **(84)**, 1-Hexacosanol **(85)**, Hexadecanol **(86)**, Zarzissine **(87)**, Choline **(88)**, Allantoin **(89)**, Adenine **(90)**, Hypoxanthine **(91)**, Uracil **(92)**, 2.4(3H, 5H)-Pyrimidinedione **(93)**, 2,3- Dihydroxypropyl hexadecanoate **(94)**. These compounds and their corresponding structures are shown in Table 3 and Figure 8.

## 6 Pharmacological activities

**FIGURE 10:**
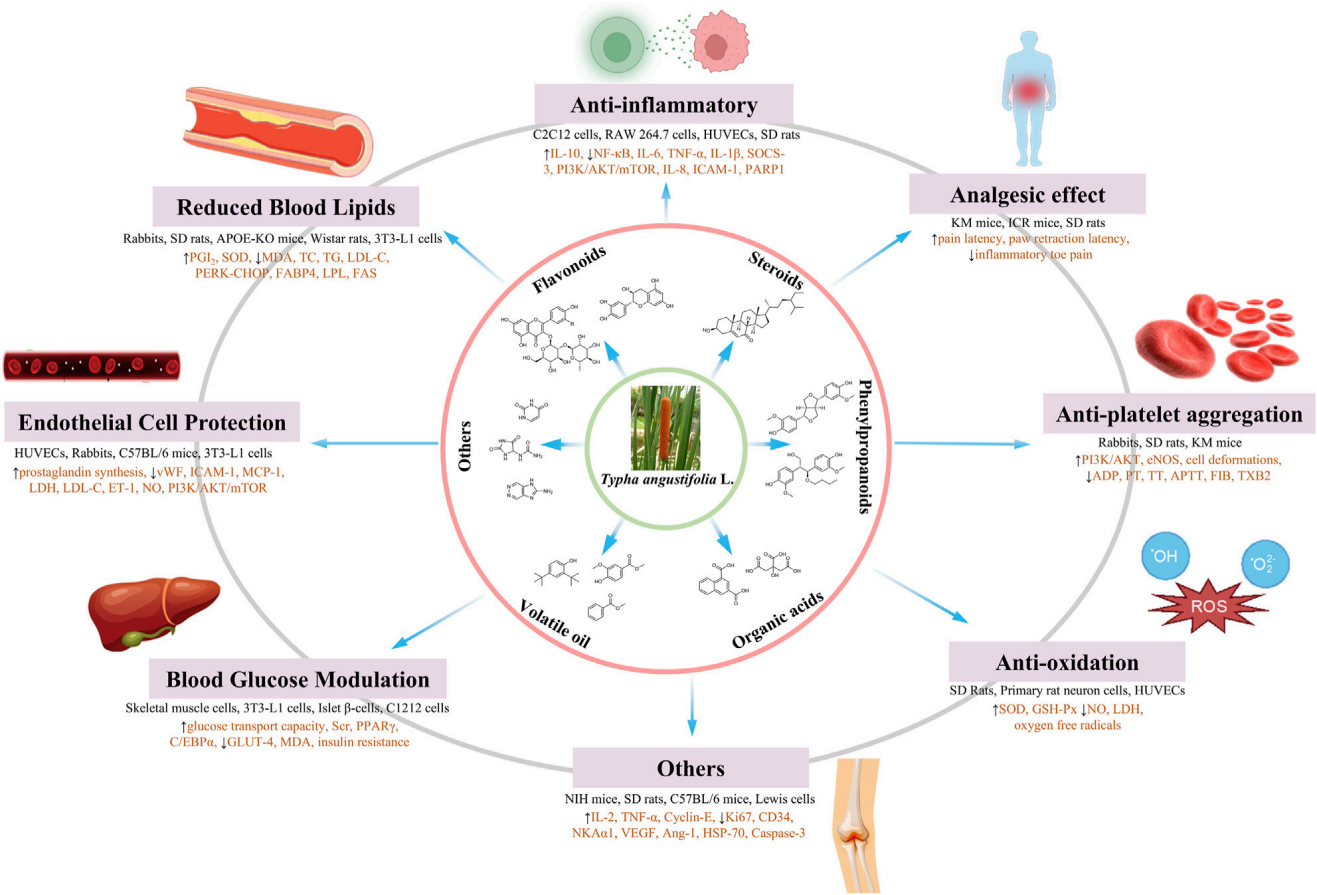
Comprehensive molecular mechanism and main components of *Typha angustifolia* L.

Given its extensive utilization in ethnopharmacological contexts, *T. angustifolia* has garnered considerable attention from researchers. Over recent decades, investigations into the pharmacological activities of *T. angustifolia* have progressively unveiled its diverse therapeutic potentials, including but not limited to anti-inflammatory, analgesic, anti-platelet aggregation, anti-atherosclerosis and anti-oxidative activities. Researchers have also carried out some studies based on different *in vivo* and *in vitro* biological models to confirm these pharmacological activities. Nonetheless, it is imperative to note that these studies have not elucidated the relationship between traditional medicinal usage and pharmacological efficacy comprehensively. Moreover, the association between bioactive metabolites and *in vivo* mechanisms remains ambiguous, with scant attention paid to pharmacokinetic parameters and bioavailability. Detailed pharmacological activities and biological studies are shown in Table 4 and Figures 9, 10.

**TABLE 4:** Pharmacological activities of *Typha angustifolia*.

Active components/Plant source	Experimental object/Method	Dose/Duration	Control		Results	References
			Positive	Negative		
Total flavonoids/Dried pollen	C2C12 murine myoblasts/*In vitro*	0.5 g/L/16 h	ROS	NA	Total flavonoids of *T. angustifolia* can inhibit IL-6mRNA expression and protein secretion of C2C12 cells by inhibiting NF-κB pathway	Lou et al., 2008b
Total flavonoids/Dried pollen	SD rats/*In vivo*	200 mg/kg/28 days	Rosiglitazone	NA	Total flavonoids of *T. angustifolia* can reduce the expression of IL-6 and SOCS-3 in skeletal muscle tissue, and improve insulin sensitivity	Feng et al. (2014a)
Total flavonoids/Dried pollen	RAW 264.7 cells/*In vitro*	0.3, 0.6, 1.2 mg/kg/24 h	NA	NA	*T. angustifolia* can inhibit the activation of Akt/mTOR signal pathway, thus induce autophagy of macrophages and reduce inflammatory infiltration	Wang et al. (2017a)
Ethanol extract/Dried pollen	Human umbilical vein endothelial cells/*In vitro*	10, 50, 100 mg/L/24 h	NA	NA	*T. angustifolia* can inhibit endothelial cell injury induced by low density lipoprotein and reduce the expression of IL-8	Wang and Li (2012)
Water extract/Dried whole plant	SD rats/*In vivo*	1, 5, 10 g/kg/28 days	NA	Saline	*T. angustifolia* can inhibit PARP1/MAPK signal pathway and its protein expression, and improve the motor function of rats	Liu et al. (2021)
Water extract/Dried pollen	SD rats/*In vivo*	5, 10, 20 g/kg/24 days	Carbazochrome Salicylate	NA	*T. angustifolia* can reduce the symptoms of hematuria and proteinuria and improve the indexes of renal function in nephritic hematuria model rats	Zhang et al. (2013)
Ethanol extract/Dried pollen	RAW 264.7 cells/*In vitro*	50 mg/mL/2 h	NA	NA	*T. angustifolia* can inhibit the expression of iNOS and COX-2, decreases the expression of IL-1β, IL-6 and TNF-α	Chen et al. (2021)
Water extract/Dried pollen	SD rats/*In vivo*	50, 100, 200 mg/kg/7 days	NA	Saline	*T. angustifolia* can downregulate the expression of VEGF, VEGF2 and Ang-1 in serum of rats, and inhibit the inflammatory lesion of retina	Yang et al. (2021)
Water extract/Dried pollen	SPF mice/*In vivo*	0.01 mL/g/30 min	Morphine	NA	*T. angustifolia* can inhibit the pain induced by tartaric acid and hot plate in mice	Ge et al. (2002)
Water extract/Dried pollen	SD rats/*In vivo*	20, 50, 70 mg/mL/60 min	NA	Saline	*T. angustifolia* can shorten the latency of paw withdrawal reaction in rats and has a significant effect on pain regulation	Zhang et al. (2017)
Water extract/Dried pollen	SD rats/*In vivo*	20, 50, 70 mg/mL/60 min	NA	Saline	*T. angustifolia* can reduce the pain sensitivity of rats to nociceptive thermal and mechanical stimuli	Fu et al. (2017)
Typhaneoside/Dried pollen	ICR mice/*In vivo*	1.2 g/kg/30 min	NA	Saline	*T. angustifolia* can significantly relieve the abdominal contraction induced by acetic acid and reduce the number of twists in mice	Zeng et al. (2018)
Organic acid/Dried pollen	Rabbits/*In vivo* and *In vitro*	15, 30, 60 mg/mL/10 min	NA	Saline	*T. angustifolia* can slow down the rate of platelet aggregation, so that the speed of platelet disaggregation is relatively faster	Feng and Liu (1999)
N-butanol extract/Dried pollen	SD rats/*In vivo*	3 g/kg/10 days	YunnanBaiyao	NA	*T. angustifolia* can reduce prothrombin time, thrombin time, activated partial thromboplastin time and serum fibrinogen content in rats	Ma et al. (2010)
Water extract/Dried pollen	SD rats/*In vivo*	2, 4, 8 g/kg/7 days	Aspirin	NA	*T. angustifolia* can inhibit the formation of arteriovenous thrombus and reduce the thrombus quality and embolization rate	Zhao and Zhu (2011)
Water extract/Dried pollen	SD rats/*In vivo*	1.2 g/kg/7 days	YunnanBaiyao	NA	*T. angustifolia* can improve the whole blood viscosity, shorten the clotting time and reduce the content of fibrinogen in blood	Kong et al. (2011)
Total flavonoids/Dried pollen	New Zealand Rabbits/*In vivo*	0.2, 0.5, 0.8 g/kg/7 days	Tanshinone	Saline	*T. angustifolia* can significantly reduce rabbit whole blood viscosity, reduce hematocrit and inhibit platelet aggregation	Yang and Li (2012)
Ethyl acetate extract/Dried pollen	New Zealand Rabbits and KM mice/*In vivo* and *In vitro*	15, 30, 60 mg/kg/7 days	Aspirin	NA	The Ethyl acetate extract of *T. angustifolia* can inhibit thrombus formation in mice and rabbit platelet aggregation induced by arachidonic acid, collagen and thrombin	He et al. (2014)
Water extract/Dried pollen	SD rats/*In vivo*	1.8, 3.6, 7.2 g/kg/7 days	NA	Saline	*T. angustifolia* can reduce the contents of TXB2 and 6-keto-PGF1 α in plasma of blood stasis model rats, and improve the rate of platelet aggregation	Shen et al. (2015)
Ethanol extract/Dried pollen	SD rats/*In vivo* and *In vitro*	200 mg/L/10 min	Hirudin	Saline	*T. angustifolia* can antagonize platelet aggregation induced by ADP and thrombin and decrease the level of intracellular calcium in rats	Wang et al. (2017b)
Containing serum/Dried pollen	Rat aortic endothelial cells/*In vitro*	160 μg/mL/20 days	Vitamin E	NA	*T. angustifolia* can promote endothelial cells to synthesize PGI_2_ and reduce the production of lipid peroxides	Qiang et al. (1987)
Water extract/Dried pollen	Japanese White Rabbits/*In vivo*	0.1, 0.2 g/mL/84 days	Tanshinon	NA	*T. angustifolia* can reduce the levels of serum total cholesterol, triglyceride and LDL-C in hyperlipidemic rabbits	Tao and Li (2004)
Water extract/Dried pollen	Quail/*In vivo*	1, 2, 4 g/kg/28 days	Zhibituo Pill	NA	*T. angustifolia* can reduce the levels of total cholesterol and triglyceride in plasma of hyperlipidemic quail	Zhou et al. (2006)
Total flavonoids/Dried pollen	APOE-KO mice/*In vivo*	0.2, 0.4, 0.8 g/kg/84 days	NA	Saline	*T. angustifolia* can relieve the stress of aortic endoplasmic reticulum and significantly reduce the level of blood lipids in atherosclerotic mice	Mo (2019)
Water extract/Dried pollen	Wistar rats/*In vivo*	2, 4, 8 g/kg/56 days	NA	Saline	*T. angustifolia* can regulate the disorder of lipid metabolism and upregulate the expression of LDLmRNA in liver tissue	Jiang et al. (2008)
Total flavonoids/Dried pollen	3T3-L1 cells/*In vitro*	0.025, 0.05, 0.1, 0.2, 0.4 g/L/24 h	Rosiglitazone	NA	*T. angustifolia* can significantly increase the glucose uptake of 3T3-L1 adipocytes and reduce FFA overflow	He et al. (2008)
Total flavonoids/Dried pollen	3T3-L1 cells/*In vitro*	0.2 g/L/24 h	Rosiglitazone	NA	*T. angustifolia* can improve insulin resistance by up-regulating the expression of PPAR and its related receptors in 3T3-L1 adipocytes	He et al. (2008)
Typhaneoside/Dried pollen	C57BL/6 mice/*In vivo* and *In vitro*	30 mg/kg/56 days	NA	NA	*T. angustifolia* can improve lipid accumulation and inflammatory damage caused by high-fat diet by activating the expression of FXR.	Zheng et al. (2023)
Water extract/Dried pollen	Japanese White Rabbits/*In vivo*	8 g/kg/35 days	NA	Saline	*T. angustifolia* can reduce vascular endothelial injury in rabbit model of hyperlipidemia by improving hemorheology	Zhang et al. (2003)
Ethanol extract/Dried pollen	Human umbilical vein endothelial cells/*In vitro*	50, 100, 150 μg/mL/4 h	Estradiol	NA	*T. angustifolia* can protect vascular endothelial cells from hypoxia injury by inhibiting the production of NO and ET-1	Lin et al. (2011)
Total flavonoids/Dried pollen	Skeletal muscle cells/*In vitro*	0.125, 0.25, 0.5, 1.0, 2.0 g/L/24 h	Rosiglitazone	NA	*T. angustifolia* can improve the insulin resistance of skeletal muscle cells induced by palmitic acid and increase its glucose transport efficiency	Lou et al. (2008b)
Total flavonoids/Dried pollen	Skeletal muscle cells/*In vitro*	0.1, 0.25, 0.5 g/L/8, 16, 24 h	Insulin	NA	*T. angustifolia* can increase glucose consumption of skeletal muscle cells and promote the expression of Src	Liu et al. (2014)
Total flavonoids/Dried pollen	3T3-L1 cells/*In vitro*	0.05, 0.1, 0.2, 0.4, 0.8 g/L/12, 24, 48, 72 h	Rosiglitazone	NA	*T. angustifolia* can increase the expression of PPARγ and C/EBP and antagonize insulin resistance induced by fatty acids	Fu et al. (2010)
Total flavonoids/Dried pollen	Islet β-cells/*In vitro*	0.125, 0.25, 0.5, 0.75, 1.0 mM/16, 24, 48 h	NA	NA	*T. angustifolia* can mitigate the damage to islet β-cells induced by fatty acids by reducing endoplasmic reticulum stress and oxidative stress products	Feng et al. (2015)
Water extract/Dried pollen	SD Rats/*In vivo*	0.2, 0.4 g/kg/15 days	NA	Saline	*T. angustifolia* can reduce the production of MDA and upregulate the activity of SOD in brain tissue after ischemia-reperfusion	Wang et al. (2003)
Water and ethanol extract/Dried pollen	Primary rat neuron cells/*In vitro*	0.25, 0.5, 1.25 mg/mL/48 h	NA	NA	*T. angustifolia* can upregulate the activities of GSH-Px and SOD in neuron cells, and decrease the content of MDA.	Chen et al. (2006)
Ethanol extract/Dried pollen	Human umbilical vein endothelial cells/*In vitro*	2.8–70 mM/24 h	NA	NA	*T. angustifolia* can reduce the content of MDA and NO and increase the activity of SOD in LPS-induced inflammation model *in vitro*	Chen et al. (2017)
Granule preparation/Dried whole plant	NIH mice/*In vivo*	30, 150 mg/kg/50 days	Prostat	NA	*T. angustifolia* can reduce the level of serum testosterone and inhibit prostatic hyperplasia induced by testosterone propionate in mice	Qin et al. (2017)
Granule preparation/Dried whole plant	NIH mice/*In vivo*	30 mg/kg/30 days	Prostat	NA	*T. angustifolia* can reduce the level of serum testosterone and inhibit prostatic hyperplasia induced by testosterone propionate in mice	Zhu et al. (2018)
Water extract/Dried pollen	Isolated uterine smooth muscle from SD rats/*In vitro*	2, 4, 8, 12 mg/mL/10 min	Verapamil	NA	*T. angustifolia* can enhance the muscle tension and prolong the contraction time of isolated rat uterine smooth muscle	Lai et al. (2016)
Water extract/Dried pollen	Human vascular smooth muscle cells/*In vitro*	0.1 g/mL/24, 48, 72 h	Ouabain	NA	*T. angustifolia* can inhibit vascular smooth muscle calcification by blocking osteopontin signal pathway	Lou et al. (2017)
Water extract/Dried pollen	C57BL/6 mice and Lewis lung carcinoma cells/*In vivo* and *In vitro*	50, 100, 200 mg/kg/14 days	Fluorouracil	Saline	*T. angustifolia* can inhibit the growth of Lewis lung carcinoma cells, arrest the tumor cells in G1 phase and induce their apoptosis	Chen et al. (2008a)
Water extract/Dried whole plant	C57BL/6 mice and Lewis lung carcinoma cells/*In vivo* and *In vitro*	50, 100, 200 mg/kg/10 days	Fluorouracil	Saline	*T. angustifolia* can significantly improve the proliferation of spleen lymphocytes and the levels of IL-2 and TNF- α in serum	Li et al. (2011)
Water extract/Dried pollen	C57BL/6 mice/*In vivo*	0.35 g/mL/14 days	NA	NA	*T. angustifolia* can mitigate ischemia-reperfusion-induced brain injury by regulating the expression of Parkin and Cyclin E	Yuan and Xia (2014)

### 6.1 Anti-inflammatory

The total flavonoids of *T. angustifolia* exhibit inhibitory effects on the expression of IL-6 mRNA and the secretion of protein in C2C12 skeletal muscle cells cultured with palmitic acid. This inhibition contributes to the alleviation of skeletal muscle inflammation and the amelioration of insulin resistance within the skeletal muscle, implying a potential mechanism linked to the regulation of glucose transport efficiency and the suppression of the NF-κB pathway (Lou et al., 2008b). Further investigations into the anti-inflammatory properties of *T. angustifolia* have revealed that its total flavonoids can diminish the levels of IL-6 and TNF-α in the serum of type 2 diabetic rats induced by a high-fat diet and low-dose Streptozocin (Feng et al., 2014a). Additionally, they downregulate the content of SOCS-3 by modulating the JAK/STAT pathway, thus mitigating chronic inflammation within the circulatory system. *Typha angustifolia* also exhibited an inhibitory effect on the PI3K/AKT/mTOR pathway *in vitro*. This effect is significant as it reduces inflammation by inducing macrophage autophagy and inhibiting the secretion of the inflammatory factor IL-10 (Wang D. et al., 2017).

*Typha angustifolia* can inhibit the damage of vascular endothelial cells induced by Ox-LDL, reduce the intercellular adhesion and inhibit the further occurrence of inflammatory reaction by down-regulating the expression of IL-8 mRNA (Wang and Li, 2012). Liu et al. (2021) reported that *T. angustifolia* can suppress the inflammatory response by inhibiting the expression of PARP1 and MAPK, consequently reducing lymphocyte infiltration and enhancing motor function in an acute spinal cord injury rat model. Zhang et al. (2013) used the combination of immunogenic bovine serum albumin, CCl4 and LPS to induce a rat model of nephritis hematuria, and found that *T. angustifolia* could significantly reduce the number of malformed red blood cells in the urine of rats, the contents of urinary protein and creatinine, and the value of serum urea nitrogen. It can increase the Endogenous creatinine clearance rate of serum total protein and endogenous creatinine clearance rate, and has a certain protective effect on renal function. Another study also reported that *T. angustifolia* can effectively inhibit the expression of iNOS and COX-2 in LPS-induced RAW264.7 cells, and then downregulate the content levels of inflammatory factors IL-1β, IL-6 and TNF-α (Chen et al., 2021). Analysis of the active metabolites within *T. angustifolia* extracts indicated that organic acids and flavonoids may constitute its primary anti-inflammatory substance foundation. It is worth mentioning that *T. angustifolia* can also protect the blood-retinal barrier function by inhibiting pro-inflammatory cytokines such as TNF-α and ICAM-1. This action reduces the excessive release of NO, thereby alleviating retinal inflammation and exerting a protective effect on retinal microangiopathy caused by diabetes (Yang et al., 2021).

### 6.2 Analgesic effect

Researchers observed the analgesic effect of *T. angustifolia* by plate method and writhing body method. They found that *T. angustifolia* significantly prolonged the number of foot lickings and pain latency in mice, exerting a notable inhibitory effect on pain induced by chemical and physical stimuli. Interestingly, the analgesic effect of *T. angustifolia* surpassed that of diclofenac sodium in the positive drug group (Ge et al., 2002). Zhang et al. (2017)’s study also confirmed this point, and further explored the effect of *T. angustifolia* on the regulation of pain perception in rats by using pressure plate pain measurement method, and found that the analgesic activity of *T. angustifolia* was dose-dependent, and based on the solubility of active metabolites in different organic solvents, it was speculated that flavonoids might be the main active metabolites of *T. angustifolia* for analgesia. In addition, *T. angustifolia* also exhibited an inhibitory effect on inflammatory toe pain induced by exogenous substances, significantly prolonging the paw retraction latency in inflamed rats (Fu et al., 2017). In a study utilizing an LC-MS combined effect-directed fractionation strategy, two active metabolites, Isorhamnetin-3-O-neohesperidin **(10)** and Typhaneoside **(16),** were isolated and identified from *T. angustifolia*. Both compounds demonstrated significant reductions in the number of body twists induced by acetic acid in mice, with inhibition rates of 54% and 51.8%, respectively (Zeng et al., 2019).

### 6.3 Anti-platelet aggregation

*Typha angustifolia* can inhibits platelet aggregation induced by ADP, arachidonic acid, collagen, and other inducers *in vitro*, slowing down the aggregation rate and depolymerizing the irreversible platelet aggregation, and this inhibitory effect is dose-dependent (Feng and Liu, 1999). *Typha angustifolia* can reduce the prothrombin time, thrombin time, activated partial thromboplastin time, and serum fibrinogen content in rats (Ma et al., 2010). Additionally, further animal experiments revealed that *T. angustifolia* inhibits arteriovenous anastomosis thrombosis formation in rats, decreases the rate of arterial thromboembolism induced by electrical stimulation, and exhibits a certain protective effect on endothelial cells (Zhao and Zhu, 2011). One study observed the effects of *T. angustifolia* on hemorheology indexes in rats and found that it could significantly reduce whole blood viscosity, enhance red blood cell deformations, and decrease aggregation without significantly affecting hematocrit in each dose group, indicating an improvement in blood circulation (Kong et al., 2011). Yang and Li (2012) reported similar research results, further confirming that the total flavonoids of *T. angustifolia* are active metabolites against platelet aggregation. These flavonoids improved the elevation of rabbit whole blood viscosity induced by a combination of adrenaline and ice bath, reduced the maximum platelet aggregation rate, and shortened the maximum agglutination time.

In addition to its influence on hemorheology, *T. angustifolia* can also reduce the content of TXB2 and 6-keto-PGF1α in the plasma of blood stasis model rats, thus improving the platelet aggregation rate and inhibiting thrombosis (Shen et al., 2015). He et al. (2014) reported that *T. angustifolia* significantly inhibits the formation of thromboxemia in rats and has a protective effect on vascular endothelial cells. Building on this foundation, the anti-platelet aggregation activities of different organic solvent extracts of *T. angustifolia* were systematically screened, leading to the preliminary confirmation that the active metabolites are enriched in the ethyl acetate extract of *T. angustifolia*. Furthermore, various analysis methods based on network construction and genomics have also been employed to study the anti-platelet aggregation activity of *T. angustifolia*. For instance, Wang X. et al. (2017) evaluated *T. angustifolia*‘s anti-platelet aggregation activity using a high-throughput platelet aggregation experimental system *in vitro*. They discovered that *T. angustifolia* could decrease the level of calcium ions in platelets, potentially inhibiting platelet aggregation by activating the PI3K/Akt/eNOS pathway.

### 6.4 Anti-atherosclerosis

*Typha angustifolia* effectively reduces the formation and development of atherosclerotic plaques and diminishes the lesion area through various mechanisms, including the reduction of blood lipid levels, lipid accumulation, and promotion of endothelial cell proliferation. Its anti-atherosclerotic activity primarily involved lipid reduction and endothelial cell protection.

#### 6.4.1 Reduced blood lipids

*Typha angustifolia* can promote the synthesis of prostacyclin (PGI2), reduce the production of lipid peroxides by vascular endothelial cells, reduce the levels of serum TC, LDL-C and TC/HDL-C by inhibiting intestinal absorption of exogenous cholesterol, and inhibit the increase of TXB2 content (Qiang et al., 1987; Zhang et al., 1987). In a variety of animal models, *T. angustifolia* showed significant activity in lowering blood lipids. Tao and Li (2004) reported that *T. angustifolia* could reduce the levels of total cholesterol, triglycerides, and low-density lipoprotein in the serum of a hyperlipidemia rabbit model. While the levels of TC and TG in the plasma of the hyperlipidemia quail model decreased, the levels of endothelin and nitric oxide were not significantly affected. By enhancing the function of macrophages, *T. angustifolia* could reduce the hyperlipidemia induced by high-fat diet, but had no effect on the elevation of ALT caused by high-fat (Reng et al., 1989; Zhou et al., 2006). Jiang and Huang (2009) induced hyperlipidemia model in rats by intraperitoneal injection of vitamin D and high-fat diet, and found that *T. angustifolia* could significantly reduce the content levels of TC, TG, LDL-C and NO in the serum of rats. By down-regulating the expression of PERK-CHOP pathway, *T. angustifolia* also can inhibit endoplasmic reticulum stress and reduce cell apoptosis of aortic endothelial cells in atherosclerotic mice, and reduce the average area of atherosclerotic lesions (Mo, 2019).

Additionally, it has also been observed that *T. angustifolia* can prevent excessive accumulation of intracellular cholesterol and regulate the balance of total cholesterol in the body by up-regulating the expression of density lipoprotein receptor mRNA (Jiang et al., 2008). An *in vitro* study reported that total flavonoids of *T. angustifolia* can reduce the secretion of free fatty acids in 3T3-L1 adipocytes and regulate the lipid metabolism of the cells (He et al., 2006a). Further studies found that the lipid-lowering mechanism of bulrushes involved down-regulating the expression of major transcription factors in the fat formation pathway, which could inhibit the transport, uptake, and synthesis of lipids by reducing the levels of lipid metabolizing enzymes FABP4, LPL and FAS (He et al., 2008). Moreover, Typhaneoside **(16)**, one of the active metabolites of *T. angustifolia*, can dose-dependently improve non-alcoholic fatty liver disease induced by a high-fat diet in mice. This improvement occurs through the activation of FXR expression, leading to a reduction in lipid accumulation in the liver and white adipose tissue. This demonstrates the multifaceted lipid-lowering properties of *T. angustifolia* and its active metabolites, presenting potential therapeutic avenues for combating dyslipidemia-related conditions (Zheng et al., 2023).

#### 6.4.2 Endothelial cell protection

*Typha angustifolia* has demonstrated a significant protective effect on endothelial cells in various *in vivo* animal experimental models. It enhances prostaglandin synthesis in arterial endothelial cells of spontaneously hypertensive rats, reduces the concentration of intracellular free calcium ions, improves transport efficiency, and preserves endothelial cell function, thus significantly preventing the formation of atherosclerotic plaques (Qiang et al., 1987). Zhou et al. (2006) reported similar findings, indicating that *T. angustifolia* could notably inhibit the formation of atherosclerotic lesions in dyslipidemic quail. Additionally, it also inhibits the migration of macrophages and fibroblasts into the subintima, reduces the formation of lipostriated arteriosclerosis plaques in rabbit atherosclerotic models, and mitigates risk factors associated with endothelial cell injury (Tao and Li, 2004).

*Typha angustifolia* can significantly reduce the plasma vWF content, increase the plasma NO content, enhance the endothelum-dependent relaxation response induced by acetylcholine and norepinephrine induced contraction response of thoracic aorta. It has obvious protective effect on vascular endothelial injury caused by hyperlipidemia. This underscores its pronounced protective effect against vascular endothelial injury caused by hyperlipidemia (Zhang et al., 2003). Additionally, *T. angustifolia* inhibits the upregulated expression of ICAM-1 and MCP-1 in human umbilical vein endothelial cells induced by oxidized low-density lipoprotein, leading to reduced LDH release, enhanced endothelial cell proliferation, and protection against inflammation-induced vascular endothelial injury (Wang and Huang, 2010). Further investigation into its active compounds revealed that total flavonoids are among the primary active substances of *T. angustifolia*. These flavonoids exhibit protective effects against endothelial cell damage induced by hypoxia, improving the activity of human umbilical vein endothelial cells under hypoxic conditions. Moreover, they inhibit the production of the endogenous vasoactive factor, human endothelin-1 (ET-1), and regulate the balance of NO/ET-1 (Lin et al., 2011). By activating the autophagy pathway of macrophages, total flavonoids of *T. angustifolia* can reduce the degradation of extracellular matrix and inhibit the formation and development of plaque, suggesting that the mechanism may be related to the inhibition of the activation of PI3K/AKT/mTOR pathway (Wang D. et al., 2017).

### 6.5 Blood glucose modulation

*Typha angustifolia* can enhance the glucose uptake and transport capacity of 3T3-L1 adipocytes in a dose-dependent manner (He et al., 2006b). Lou et al. (2008a) reported that *T. angustifolia* improves palmitic acid-induced insulin resistance in C2C12 skeletal muscle cells. It increases cellular glucose utilization efficiency and uptake capacity by upregulating glucose transporter (GLUT-4) expression. It can also upregulate the expression of Scr protein and promote the formation of its signal complex, and increase the glucose consumption level of skeletal muscle cells (Liu et al., 2014). Feng et al. (2012a) reported similar results, and further confirmed *in vitro* experiments that total flavonoids of *T. angustifolia* can improve skeletal muscle insulin resistance by increasing the expression of β-arrestin-2 protein in cells. Fu et al. (2010) reported that total flavonoids from *T. angustifolia* can enhance the expression of PPARγ and C/EBP-α by promoting the proliferation and differentiation of adipocytes, thus antagonizing the insulin resistance induced by fatty acids. Further studies showed that *T. angustifolia* can also improve fatty acid-induced islet beta cell damage, which may be related to the reduction of endoplasmic reticulum stress and the reduction of production of oxidative stress products (Feng et al., 2015).

### 6.6 Anti-oxidation

Wang et al. (2003) conducted an investigation using a rat model of cerebral ischemia-reperfusion injury to assess the protective effects of *T. angustifolia* on brain tissue. The findings revealed that *T. angustifolia* significantly elevated the activities of LDH and SOD in brain tissue while inhibiting lipid peroxidation damage. Similarly, Jiang and Huang (2009) also corroborated these results in a rat model of atherosclerosis, demonstrating the antioxidant activity of *T. angustifolia*. This activity notably reduced the content of MDA in rat serum and suppressed the production of oxygen free radicals within the non-enzymatic system. Chen et al. (2006) reported that co-culturing *T. angustifolia* with rat nerve cells markedly boosted the activities of GSH-Px and SOD within the cells. This led to a reduction in MDA content and mitigated brain tissue lipid peroxidation damage induced by mercury, and it also significantly enhanced the antioxidant capacity of nerve cells following mercury injury. Wang and Li (2010) confirmed through *in vitro* experiments using cultured human umbilical vein endothelial cells that *T. angustifolia* can decrease LDH and MDA activities, increase SOD activity, and rectify endothelial cell dysfunction induced by oxidized low-density lipoprotein. Furthermore, a comprehensive study evaluated the antioxidant activity of various extracts of *T. angustifolia*. It was found that both ethanol extract and water extract had significant free radical scavenging ability and antioxidant activity, which could significantly reduce the content of MDA and NO in endothelial cells under oxidative stress, and increase the content of SOD (Chen et al., 2017).

### 6.7 Others

Qin et al. (2017) reported that *T. angustifolia* can inhibit prostatic hyperplasia induced by testosterone propionate and reduce serum testosterone levels. In a mouse model of prostatic hyperplasia, Zhu et al. (2018) confirmed that *T. angustifolia* reduces the expression of Ki67 and CD_34_ antigens in prostate tissue, inhibiting pathological hyperplasia of prostate cells and reducing the aggregation of vascular endothelial cells. *Typha angustifolia* can enhance muscle tension, improve contractility, and prolong the contractility of isolated uterine smooth muscle in late pregnancy rats. This effect can be inhibited by prostaglandin synthetase inhibitors and calcium channel blockers, suggesting that the effect of *T. angustifolia* on isolated uterine smooth muscle involves various regulatory pathways (Lai et al., 2016). Additionally, *T. angustifolia* significantly inhibits the calcification of human vascular smooth muscle cells induced by ouabain. Its mechanism involves the inhibition of the NKA-α1 subunit and the blocking of the osteopontin signaling pathway (Lou et al., 2017).

The water extract of *T. angustifolia* inhibits the growth of Lewis lung cancer tumors in mice, increases serum levels of IL-2 and TNF-α, arrests tumor cells in the G_1_ phase, and induces tumor cell apoptosis (Chen et al., 2008). Additionally, tetrazole salt assay results demonstrate that *T. angustifolia* improves spleen lymphocyte proliferation in tumor-bearing mice (Li et al., 2011). *Typha angustifolia* exhibits a protective effect on retinal tissue lesions caused by hyperglycemia by down-regulating the expression of VEGF and Ang-1 and improving fasting blood glucose levels (Yang et al., 2021). Several studies have reported that *T. angustifolia* also mitigates brain tissue injury caused by ischemic reperfusion, protecting neuronal function through the regulation of proteins such as HSP-70, Caspase-3, Parkin, and Cyclin-E (Xia et al., 2008; Xia et al., 2009; Yuan and Xia, 2014). This inhibition of neuronal apoptosis and impact on mitochondrial autophagy suggest potential treatment avenues for ischemic encephalopathy and the development of novel therapeutics.

## 7 Quality control

In the 2020 edition of the Chinese Pharmacopoeia, the quality evaluation method for Typha purpurea was standardized, with isorhamnetin-3-O-neohesperidin and *T. angustifolia* serving as the index metabolites for determining its content. However, the current quality evaluation methods lack comprehensiveness, relying solely on two chemical metabolites as quality evaluation indices weakens the correlation with drug activity. Furthermore, specific identification methods and quantitative standards are lacking, it is necessary to further establish a scientific and reasonable quality evaluation model based on modern technology to guide the production, circulation, and use of *T. angustifolia* more effectively.

At present, HPLC, HPCE, LC-MS/MS, GC-MS and other high-efficiency chromatography-mass spectrometry tools are often used in the quality control research of *T. angustifolia* for qualitative and quantitative analysis of active metabolites. Wen et al. (2006) established the HPLC method for the determination of total flavonoids content in *T. angustifolia* for the first time, and ensured a good linear relationship between its index metabolites, with good repeatability and high recovery rate. Ma et al. (2014) optimized the extraction process of total flavonoids from *T. angustifolia*, elution and separation of total flavonoids with macroporous resin, and further established a stable and simple purification process. Zhang et al. (2019) established a method for simultaneous determination of Isorhamnetin-3-O-neohesperidin **(10)** and Typhaneoside **(16)** in *T. angustifolia*. Compared with the traditional method, the peak time is shorter and the two metabolites can be effectively separated. Ge and Jia (2011) established an LC-MS/PAD method for the detection of Auramine O in *T. angustifolia*, which can effectively distinguish whether the botanical drugs have been illegally stained.

Fingerprints, multivariate statistical analysis and pharmacokinetics of traditional Chinese medicine are also widely used in the process of identification and separation of index metabolites. For the first time, Lu et al. (2018) tested the content of index metabolites in *T. angustifolia* at different mature stages, and the results showed that the active metabolites were the highest in *T. angustifolia* picked in the half flowering stage. Based on the UPLC method, Tang et al. (2022) established the traditional Chinese medicine fingerprint of *T. angustifolia* and the content determination method of eight main metabolites. A total of 15 common peaks were identified in the UPLC fingerprint, and the similarity of different batches of medicinal materials was greater than 0.900. Li et al. (2015) simultaneously determined the total content of five main index metabolites in 16 batches of *T. angustifolia* decoction pieces by HPLC, and established a quality characterization and correlation analysis method based on this.

A variety of emerging technologies play an important role in the quality control research of *T. angustifolia* and have made great progress in recent years, such as *in vitro* and *in vivo* quantitative studies through the combination of multiple chromatographic technologies, analysis of the enrichment mode of active substances based on metabolomics, and prediction of active substances through the establishment of network pharmacological models. In order to establish a scientific and sound quality evaluation system, we still need to conduct in-depth analysis of chemical information and pharmacodynamic results with statistical differences on the basis of existing research, and find chemical substances with high correlation with pharmacological activity to ensure the safety and effectiveness of clinical use.

## 8 Conclusion

*Typha angustifolia* has a long history of application and is widely used the treatment of dysmenorrhea, irregular menstruation, abdominal pain, trauma bleeding, soft tissue contusion, hematochezia, hematuria, and other hemorrhagic diseases. It also serves as a resource-rich aquatic cash crop, finding extensive applications in medicine, weaving, papermaking, and the food industry. With its high medicinal value, economic potential, and bioavailability, it holds significant importance. Currently, we have reviewed the botany, traditional uses, phytochemistry, pharmacological activities, and quality control of *T. angustifolia*. The paper then delves into future research directions and focal points, considering the following aspects:

Numerous studies have confirmed that flavonoids serve as the foundation for many pharmacological activities, including anti-inflammatory, analgesic, and lipid-lowering effects. Given their promising biological activity, the quest for novel flavonoid compounds from *T. angustifolia* and exploring the correlation between their structural characteristics and efficacy represent significant research endeavors and future focuses. However, current studies primarily focus on discovering and isolating new active metabolites, often remaining at the stage of crude extract analysis. Reports on potential biological activities of new compounds are scarce. In addition, it is essential that future studies address current methodological limitations by adopting standardized bioassay protocols to rigorously validate the pharmacological efficacy of these compounds. Thus, conducting bioactivity-driven structure-activity studies on newly isolated compounds is crucial. This approach can elucidate the internal relationship between active metabolites and pharmacological activities, guiding future research directions on *T. angustifolia*‘s active metabolites, and advancing the research and development of innovative drugs.

Systematic research into the pharmacological activity of *T. angustifolia* is crucial for unlocking its medicinal potential. This medicinal plant exhibits various pharmacological activities, including anti-inflammatory, analgesic, anti-platelet aggregation, anti-atherosclerosis, and anti-oxidation properties. However, existing studies have not delved deeply into the specific action links and mechanisms of *T. angustifolia in vivo*, nor have they thoroughly explored its relationship with disease targets. The focus of traditional plant research is the rapid and accurate discovery of pharmacological effector substances. However, the current study of the pharmacological active metabolites of *T. angustifolia* primarily revolves around extracts, particularly total flavonoid extracts, with a notable absence of in-depth exploration into monomer compounds. Moreover, future studies should critically address these limitations by integrating advanced *in vivo* models and mechanistic analyses to validate pharmacological effects and elucidate disease target interactions. Therefore, it is necessary to further study the correlation between the pharmacological activities and chemical metabolites of *T. angustifolia* by combining related techniques such as pharmacokinetics and network pharmacology. This will lead to a more comprehensive understanding of *T. angustifolia*‘s medicinal properties and facilitate the development of novel therapeutic interventions.

*Typha angustifolia* has traditionally been utilized in treating hemorrhagic diseases, particularly those stemming from local thrombosis and impaired blood microcirculation. This is maybe attributed to its anti-platelet aggregation activity, indicating potential for development as a novel antithrombotic drug. However, existing studies predominantly focus on *T. angustifolia*‘s effect on blood coagulation-related indices, with limited exploration into its specific mechanism influencing blood coagulation function. Future studies should critically evaluate these mechanisms using comprehensive *in vitro* and *in vivo* models to rigorously validate its antithrombotic potential. This underscores the need for robust clinical research validation and evidence to support traditional uses of *T. angustifolia*. Hence, we need to pay attention to the role of traditional Chinese medicine theory in guiding the use and development of drugs. In order to better develop and utilize the medicinal resources of *T. angustifolia*, it is necessary to establish a close relationship with traditional medical knowledge, and further strengthen the existing pharmacological activity research on this basis.

The quality control index of *T. angustifolia* is still relatively simple, the identification of active metabolites is blind, and there is no comprehensive quality evaluation system. In the future research, based on the existing active metabolite data, combined with a variety of emerging technologies, in-depth analysis of chemical information and pharmacodynamics results with statistical differences should be carried out, further digging related quality markers and improving the quality evaluation system of *T. angustifolia*. In assessing the quality of medicinal materials comprehensively, it's essential to emphasize clinical relevance in terms of safety and effectiveness. Establishing an overall quality evaluation method should prioritize active metabolite content. Notably, T. angustifolia contains numerous volatile metabolites, which may lose their activity due to external factors like light, humidity, temperature, and oxidation. Hence, this requires us to further develop quality control methods that can monitor the production and content changes of extracts over time based on the fingerprint of existing compounds or fractions, so as to improve the stability of the quality of medicinal materials.

In conclusion, *T. angustifolia* is a traditional medicinal plant with considerable development potential and research value. It is our hope that this paper can serve as a valuable reference for all researchers interested in *T. angustifolia*.

## Abbreviations

AKT, protein kinase B; ALT, glutamic-pyruvic transaminase; Ang-1, Angiopoietins-2; APTT, activated partial thromboplastin time; Caspase-3, cysteine protease protein-3; CHOP, CCAAT/enhancer binding protein homologous protein; COX-2, cyclooxygenase-2; ET-1, endothelin-1; FABP4, fatty acid-binding protein; FAS, fatty acid synthase; FIB, fibrinogen; FXR, farnesoid X receptor; GSH-Px, glutathione peroxidase; ICAM-1, intercellular cell adhesion molecule-1; JAK, Janus Kinase; LDH, lactate dehydrogenase; LDL-C, low-density lipoprotein cholesterol; LPL, lipoprotein lipase; MAPK, mitogen-activated protein kinase; MCP-1, monocyte chemoattractant protein-1; MDA, malonaldehyde; mTOR, mammalian target of rapamycin; PARP1, Poly ADP-ribose polymerase-1; PERK, protein kinase R-like endoplasmic reticulum kinase; PGI2, prostaglandin-2; PI3K, phosphatidylin-ositol-3-kinase; PT, prothrombin time; STAT, signal transducerand activator of transcription; TC, total cholesterol; TG, triglyceride; TT, thrombin clotting time; TXB2, thromboxane; VEGF, vascular endothelial growth factor; vW, von Willebrand factor.
